# Palmitoyl Transferase FonPAT2-Catalyzed Palmitoylation of the FonAP-2 Complex Is Essential for Growth, Development, Stress Response, and Virulence in Fusarium oxysporum f. sp. *niveum*

**DOI:** 10.1128/spectrum.03861-22

**Published:** 2022-12-19

**Authors:** Xiaohui Xiong, Yizhou Gao, Jiajing Wang, Hui Wang, Jiajun Lou, Yan Bi, Yuqing Yan, Dayong Li, Fengming Song

**Affiliations:** a Zhejiang Provincial Key Laboratory of Biology of Crop Pathogens and Insects, MARA Key Laboratory of Molecular Biology of Crop Pathogens and Insects, Institute of Biotechnology, College of Agriculture and Biotechnology, Zhejiang University, Hangzhou, People’s Republic of China; Beijing Forestry University

**Keywords:** *Fusarium oxysporum* f. sp. *niveum*, protein palmitoyl transferase (PAT), protein palmitoylation, AP-2 complex, virulence, watermelon

## Abstract

Protein palmitoylation, one of posttranslational modifications, is catalyzed by a group of palmitoyl transferases (PATs) and plays critical roles in the regulation of protein functions. However, little is known about the function and mechanism of PATs in plant pathogenic fungi. The present study reports the function and molecular mechanism of FonPATs in Fusarium oxysporum f. sp. *niveum* (*Fon*), the causal agent of watermelon Fusarium wilt. The *Fon* genome contains six *FonPAT* genes with distinct functions in vegetative growth, conidiation and conidial morphology, and stress response. FonPAT1, FonPAT2, and FonPAT4 have PAT activity and are required for *Fon* virulence on watermelon mainly through regulating *in planta* fungal growth within host plants. Comparative proteomics analysis identified a set of proteins that were palmitoylated by FonPAT2, and some of them are previously reported pathogenicity-related proteins in fungi. The FonAP-2 complex core subunits FonAP-2α, FonAP-2β, and FonAP-2μ were palmitoylated by FonPAT2 in *vivo*. FonPAT2-catalyzed palmitoylation contributed to the stability and interaction ability of the core subunits to ensure the formation of the FonAP-2 complex, which is essential for vegetative growth, asexual reproduction, cell wall integrity, and virulence in *Fon*. These findings demonstrate that FonPAT1, FonPAT2, and FonPAT4 play important roles in *Fon* virulence and that FonPAT2-catalyzed palmitoylation of the FonAP-2 complex is critical to *Fon* virulence, providing novel insights into the importance of protein palmitoylation in the virulence of plant fungal pathogens.

**IMPORTANCE** Fusarium oxysporum f. sp. *niveum* (*Fon*), the causal agent of watermelon Fusarium wilt, is one of the most serious threats for the sustainable development of the watermelon industry worldwide. However, little is known about the underlying molecular mechanism of pathogenicity in *Fon*. Here, we found that the palmitoyl transferase (*FonPAT*) genes play distinct and diverse roles in basic biological processes and stress response and demonstrated that FonPAT1, FonPAT2, and FonPAT4 have PAT activity and are required for virulence in *Fon*. We also found that FonPAT2 palmitoylates the core subunits of the FonAP-2 complex to maintain the stability and the formation of the FonAP-2 complex, which is essential for basic biological processes, stress response, and virulence in *Fon*. Our study provides new insights into the understanding of the molecular mechanism involved in *Fon* virulence and will be helpful in the development of novel strategies for disease management.

## INTRODUCTION

The soilborne fungus Fusarium oxysporum, as one of top 10 fungal pathogens (1), causes vascular Fusarium wilt in a wide variety of crops, resulting in significant losses to agricultural production worldwide (2, 3). F. oxysporum has been divided into more than 100 *formae speciales* based on their specificity on different host species (4–6). Over the last 2 decades, genomics studies have provided novel insights into the molecular mechanism of pathogenicity in F. oxysporum (7–10). In the long arms race with its host plants, F. oxysporum has evolved a series of complicated mechanisms and strategies for infection and causing diseases; for example, F. oxysporum not only secretes various virulence factors such as effectors and cell wall-degrading enzymes to function in pathogenicity but also responds to extrinsic stresses from the environment and the hosts (5, 11–15). However, a global view of the molecular mechanism and regulatory network governing the virulence of F. oxysporum on different host plants remains to be established through functional characterization of novel pathogenicity genes.

Posttranslational modifications, e.g., phosphorylation, ubiquitination, glycosylation, and lipidation, play key roles in regulating the function of proteins, and many of them are involved in development, stress response, and host infection of plant-pathogenic fungi (16). Palmitoylation, also called *S*-palmitoylation or *S*-acylation, is achieved by covalently adding lipid molecules to protein targets and thus affects protein functions, e.g., membrane transport, stability, and interaction with other proteins (17–21). Palmitoylation is a reversible posttranslational lipidation that is catalyzed oppositely by a group of palmitoyl transferases (PATs) and depalmitoylation enzymes, including acyl protein thioesterases (APTs) and the alpha/beta hydrolase domain-containing protein 17 family of hydrolases (20, 22).

PATs are characterized by four to six transmembrane domains (TMDs) and a cysteine-rich domain (CRD) with an Asp-His-His-Cys (DHHC) motif that is necessary for catalytic activity (19, 23, 24). PATs are encoded by a relatively small gene family, e.g., 7 PATs identified in yeast (25), 23 in mammals (26), and 24 in Arabidopsis thaliana (27). In yeast, the functions and substrates for ScAkr1, ScErf2, Swf1, and Pfa4 have been well studied and characterized (17, 28). In Cryptococcus neoformans, deletion of *Pfa4*, which is required for the palmitoylation of Ras1 and important for Ras1 membrane localization, resulted in impaired growth and reduced virulence (29, 30). In the human-pathogenic filamentous fungus Aspergillus fumigatus, treatment with the palmitoylation inhibitor 2-bromopalmitate or mutation of the highly conserved palmitoylation motif disrupted normal AfRasA plasma membrane localization (31) and led to defects in hyphal morphogenesis, cell wall formation, and virulence (32). A. fumigatus AfAkrA and Aspergillus nidulans AnAkrA, the yeast ScAkr1p homolog, played an important role in regulating [Ca^2+^]_c_ homeostasis, and deletion of *akrA* resulted in marked defects in hyphal extension and conidiation, especially under low-calcium conditions (33). However, the function and mechanism of PATs in plant-pathogenic fungi remain elusive.

The cargo adaptor protein AP-2 complex is a heterotetrameric endocytic cargo-binding adaptor consisting of four subunits (α, β, μ, and σ) (34). In yeast, the AP-2 complex is required for vegetative growth and polarized cell responses, and the subunit α (Apl3p) played important roles in dynamics of endocytic sites, morphogenesis, and cell wall integrity (35–38). In Candida albicans, deletion of AP-2 complex subunits resulted in defects in hyphal growth, polarized morphogenesis, and cell wall organization (36, 39). In Aspergillus, the AP-2 complex plays a specialized clathrin-independent role in apical endocytosis and polar growth through interacting with endocytic markers and membrane lipid flippases (40). In the wheat scab fungus Fusarium graminearum, the AP-2 complex was found to be required for hyphal polarized growth, polar localization of FgDnfA and FgDnfB, and full virulence (41), and the subunit FgAP-2μ was involved in cell wall integrity via interacting with FgWsc2B (42).

Watermelon Fusarium wilt, caused by F. oxysporum f. sp. *niveum* (*Fon*), is one of the most devastating fungal diseases (43). It was previously found that an effector, FonSIX6, a CCR4-Not complex subunit, FonNOT2, and a nucleotide sugar transporter, FonNst2, are essential for virulence in *Fon* (44–46). The present study aimed to investigate the involvement of protein palmitoylation in *Fon* virulence and revealed that FonPAT1, FonPAT2, and FonPAT4 are required for virulence in *Fon*. A set of proteins, including the FonAP-2 complex subunits FonAP-2α, FonAP-2β, and FonAP-2μ, were palmitoylated by FonPAT2. The FonPAT2-catalyzed palmitoylation of FonAP-2α, FonAP-2β, and FonAP-2μ ensured the stability and interactions of the subunits and was essential for *Fon* virulence. These findings demonstrate the importance of FonPAT2-catalyzed protein palmitoylation in *Fon* virulence and thus provide novel insights into the regulatory function of protein palmitoylation in virulence of plant fungal pathogens.

## RESULTS

### Characterization of *FonPAT* genes in *Fon* and generation of *FonPAT* deletion mutants.

Due to the unavailability of the genome sequence information for *Fon*, a blastp search was performed against the genome sequence of Fusarium oxysporum f. sp. *lycopersici*, a closely related formal species within the F. oxysporum species complex, using yeast PATs as queries. This search identified 6 putative *PAT* genes in *Fon*, FOXG_11304, FOXG_06235, FOXG_07613, FOXG_02420, FOXG_03335, and FOXG_03646 (see Fig. S1A in the supplemental material). The open reading frames (ORFs) of these *PAT* genes were amplified with predicted sizes from *Fon* and named *FonPAT1* to *FonPAT6* (see Fig. S1A). FonPAT proteins harbor a cysteine-rich domain with a conserved DHHC motif (DHHC-CRD) and a transmembrane region (see Fig. S1B and S2A). Homologous PATs were also identified in phytopathogenic fungi such as F. graminearum and Magnaporthe oryzae, although none of them has been studied. A phylogenetic tree analysis revealed that FonPAT1 to FonPAT6 were mainly clustered into two groups with PATs from yeast, F. graminearum, and M. oryzae (see Fig. S2B). In particular, FonPAT1 was clustered with yeast ScPFA4, FonPAT2 was grouped with ScSWF1, and FonPAT4 was closely related to ScAKR1 (see Fig. S2B).

Targeted deletion mutants for each of the *FonPAT* genes were generated using the homologous recombination approach (see Fig. S3A). Fragments with different sizes in the wild type (WT) and the corresponding deletion mutants were amplified using pairs of primers located in the regions flanking the deletion targets in the *FonPAT* genes (see Fig. S3B). A single hybridizing band was detected in each of the deletion mutants in Southern blotting with the hygromycin gene fragment (*HPH*) probe, while no hybridizing band was observed in the WT (see Fig. S3C). The transcript levels of each of the *FonPAT* genes were undetectable in *ΔFonpat4* and *ΔFonpat6* and lower than 5% in *ΔFonpat1*, *ΔFonpat2*, *ΔFonpat3*, and *ΔFonpat5* (see Fig. S3D), probably due to the nonspecific amplification with the primer sets used.

### Requirement of *FonPAT1*, *FonPAT2*, and *FonPAT4* for *Fon* virulence.

**FIG 1:**
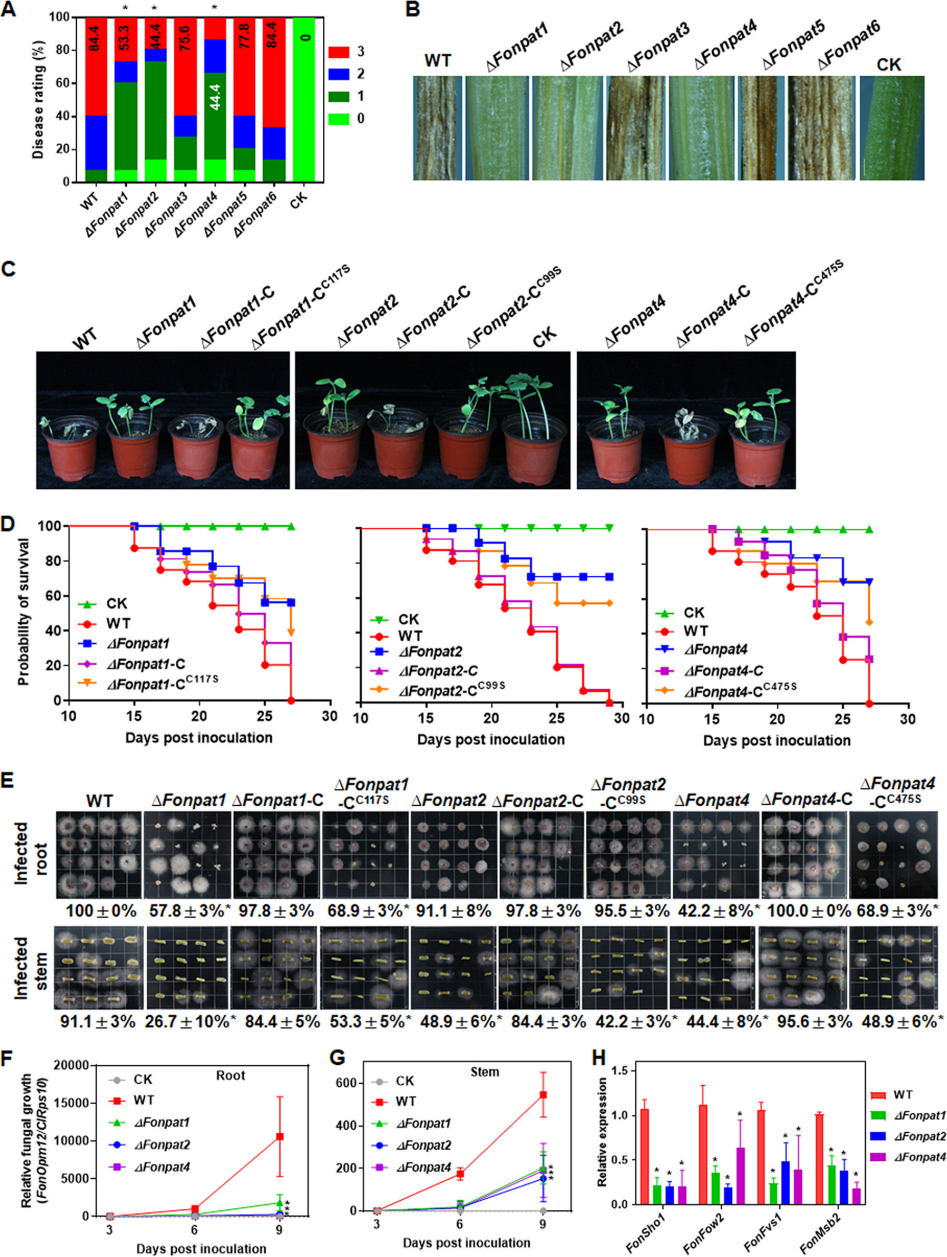
*FonPAT1*, *FonPAT2*, and *FonPAT4* were required for *Fon* virulence on watermelon. (A) Disease ratings of plants inoculated with the WT and the *ΔFonpat* mutants at 21 dpi. (B) Vascular symptoms of plants inoculated with the WT and *ΔFonpat* mutants at 14 dpi. Bar = 1 mm. CK, plants inoculated with a preparation of mung bean broth without *Fon* spores. (C) Disease phenotype of plants inoculated with the WT, *ΔFonpat1*, *ΔFonpat2*, *ΔFonpat4*, or complementation strains expressing *FonPAT1*-C, *FonPAT2*-C, *FonPAT4*-C, or DHHC-mutated variants *FonPAT1*-C^C117S^, *FonPAT2*-C^C99S^, or *FonPAT4-*C^C475S^. CK, plants inoculated with a preparation of mung bean broth without *Fon* spores. (D) Survival probability of the inoculated plants shown in panel C. (E) Fungal colonies recovered from roots and stems of plants inoculated with the WT, *ΔFonpat1*, *ΔFonpat2*, *ΔFonpat4*, or complementation strains expressing *FonPAT1*-C, *FonPAT2*-C, *FonPAT4*-C, or DHHC-mutated variants *FonPAT1*-C^C117S^, *FonPAT2*-C^C99S^, or *FonPAT4-*C^C475S^. (F and G) *In planta* fungal growth in roots and stems of plants inoculated with the WT, *ΔFonpat1*, *ΔFonpat2*, or *ΔFonpat4*. Relative fungal growth was analyzed by qPCR and is shown as ratios of *FonOpm12*/*ClRps10*. (H) Expression changes of infection-related genes in WT, *ΔFonpat1*, *ΔFonpat2*, and *ΔFonpat4* strains. Experiments were independently performed three times with similar results, and results from one representative experiment are shown in panels B, C, and E. The data presented in panels A, D to G, and H are the means ± standard deviation (SD) from three independent experiments; asterisks indicate the significant difference at *P < *0.05.

Repeated experiments showed that the disease severities of the *ΔFonpat3-*, *ΔFonpat5-*, and *ΔFonpat6-*inoculated plants were comparable to that of wild type (WT)-inoculated plants (see Fig. S4A). In contrast, >60% of the *ΔFonpat1-*, *ΔFonpat2-*, and *ΔFonpat4-*inoculated plants showed chlorosis or no evident symptom on leaves, and the disease severities were significantly reduced, leading to decreases of 31%, 40%, and 40%, respectively, compared with that of WT-inoculated plants (Fig. 1A; see also Fig. S4A). Anatomic examination revealed that the vascular systems turned brown and became necrotic in stems of WT-, *ΔFonpat3-*, *ΔFonpat5-*, and *ΔFonpat6-*inoculated plants, while the vascular systems in *ΔFonpat1-*, *ΔFonpat2-*, and *ΔFonpat4-*inoculated plants remained normal (Fig. 1B). These results indicate that *FonPAT1*, *FonPAT2*, and *FonPAT4* play important roles in *Fon* virulence, while *FonPAT3*, *FonPAT5*, and *FonPAT6* may not be involved in *Fon* virulence.

To further confirm the functions of *FonPAT1*, *FonPAT2*, and *FonPAT4* in *Fon* virulence, the disease progress and *in planta* fungal growth in *ΔFonpat1-*, *ΔFonpat2-*, or *ΔFonpat4-*inoculated plants was compared with those in WT-inoculated plants. Disease-caused death of the WT-inoculated plants first appeared at 15 days postinoculation (dpi), while the first death of the *ΔFonpat1-*, *ΔFonpat2-*, and *ΔFonpat4-*inoculated plants was delayed by 2 to ~4 days (Fig. 1C and D). At 27 dpi, 56%, 72%, and 69% of the *ΔFonpat1-*, *ΔFonpat2-*, and *ΔFonpat4-*inoculated plants, respectively, survived, while all of the WT-inoculated plants died (Fig. 1C and D). In tissue-based examination of fungal growth assays (47–49), 91.8% of root segments from *ΔFonpat2-*inoculated plants supported the fungal growth and colony formation, comparable to that (100%) in WT-inoculated plants, while 57.8% and 42.8% of root segments from *ΔFonpat1*- and *ΔFonpat4*-inoculated plants, respectively, provided the fungal growth and colony formation, significantly lower than those in WT-inoculated plants (Fig. 1E). Meanwhile, 26.7%, 48.9%, and 44.8% of stem segments from *ΔFonpat1*-, *ΔFonpat2*-, and *ΔFonpat4*-inoculated plants, respectively, supported fungal growth and colony formation, significantly lower than that (91.1%) in WT-inoculated plants (Fig. 1E). The relative fungal growth, represented as the ratio of the transcript levels of *FonOpm12* and that of watermelon *ClRps10* by quantitative PCR (qPCR), in roots and stems of the *ΔFonpat1*-, *ΔFonpat2*-, and *ΔFonpat4*-inoculated plants was significantly decreased by 83%, 97%, and 99% in roots and by 63%, 72%, and 65% in stems, respectively, compared with those in WT-inoculated plants at 9 dpi (Fig. 1F and G). The expression of *FonFvs1*, *FonMsb2*, *FonSho1*, and *FonFow2*, four previously known infection-related genes (50–52), was significantly downregulated in *ΔFonpat1*, *ΔFonpat2*, and *ΔFonpat4* (Fig. 1H). Furthermore, *ΔFonpat1* and *ΔFonpat4* lost the penetration ability against cellophane membrane, while *ΔFonpat2* retained this ability (see Fig. S4B). Complementation strains *ΔFonpat1*-C, *ΔFonpat2*-C, and *ΔFonpat4*-C were also created by introducing fragments containing the native promoters and ORFs into the corresponding mutants *ΔFonpat1*, *ΔFonpat2*, and *ΔFonpat4*, respectively. In the pathogenicity experiments, the complementation strains *ΔFonpat1*-C, *ΔFonpat2*-C, and *ΔFonpat4*-C restored the virulence, *in planta* fungal growth, expression of pathogenicity genes, and penetration ability to the WT (Fig. 1C to G). These results suggest that deletion of *FonPAT1*, *FonPAT2*, and *FonPAT4* affects the expression of pathogenicity genes and the ability of *in planta* fungal growth, thus demonstrating the importance of *FonPAT1*, *FonPAT2*, and *FonPAT4* in *Fon* virulence.

### Functions of *FonPAT* genes in basic biological processes of *Fon*.

**FIG 2:**
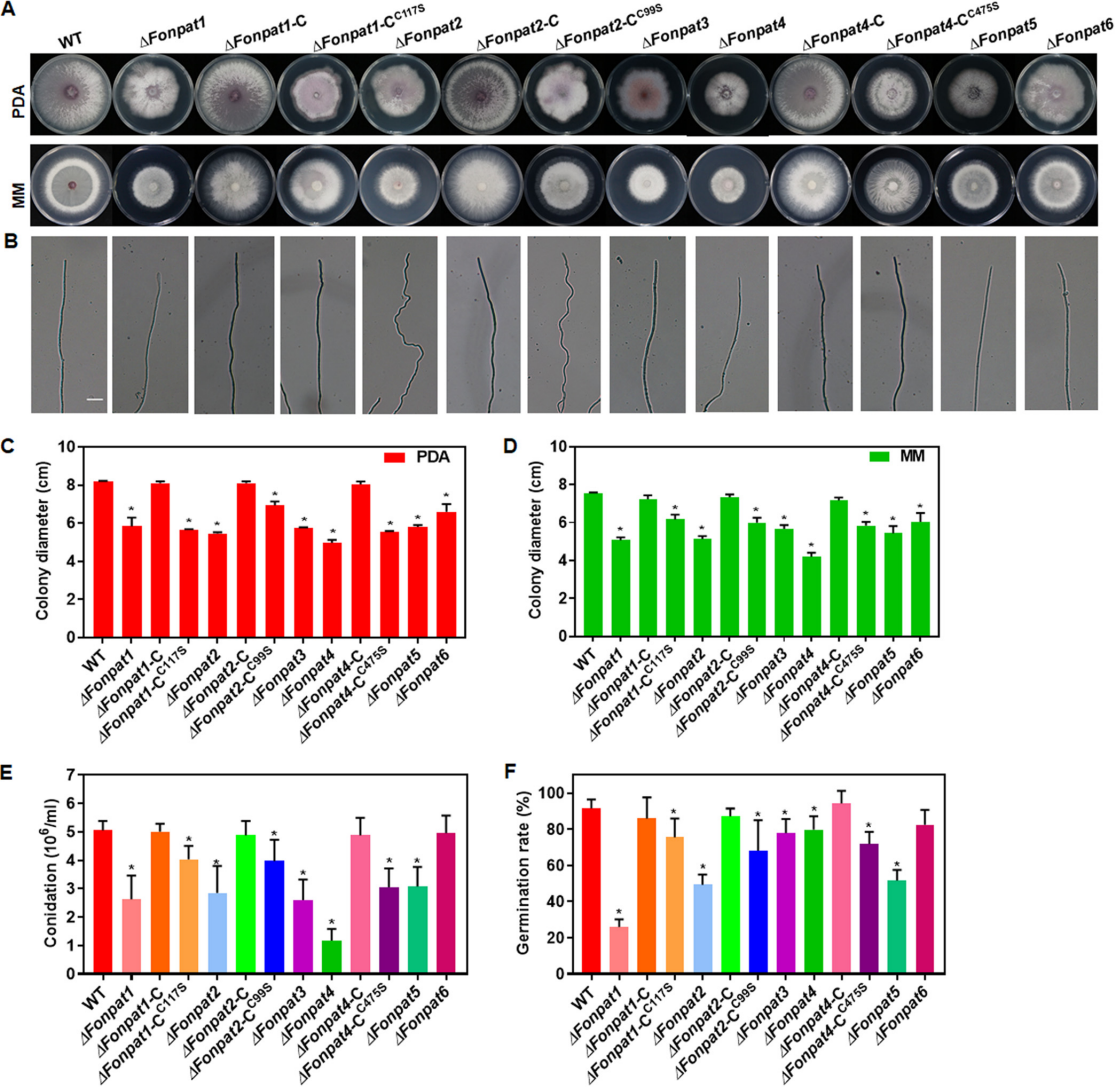
Functions of *FonPAT* genes in vegetative growth, conidiation, and spore germination of *Fon*. (A) Growth phenotype; (B) hyphal polarized growth; (C and D) colony diameters on PDA (C) and MM plates (D); (E) conidiation; (F) spore germination of the WT, deletion mutants *ΔFonpat1*, *ΔFonpat2*, *ΔFonpat3*, *ΔFonpat4*, *ΔFonpat5*, and *ΔFonpat6*, and complementation strains expressing *FonPAT1*-C, *FonPAT2*-C, *FonPAT4*-C, or DHHC-mutated variants *FonPAT1*-C^C117S^, *FonPAT2*-C^C99S^, or *FonPAT4-*C^C475S^. The indicated strains were grown on PDA or MM plates for 7 days at 26°C to estimate the mycelial growth and observe the hyphal morphology and were grown in MBL broth for 2 days at 26°C to count the macroconidia. Macroconidia were suspended in YEPD broth and incubated for 12 h to estimate the germination status by examining at least 100 macroconidia. Hyphal polarized growth was observed under a differential interference contrast microscope. Scale bar in panel B = 50 μm. Experiments were independently performed three times with similar results, and the results from one representative experiment are shown in panels A and B. The data presented in panels C to F are the means ± SD from three independent experiments; asterisks indicate the significant difference at *P < *0.05.

**FIG 3:**
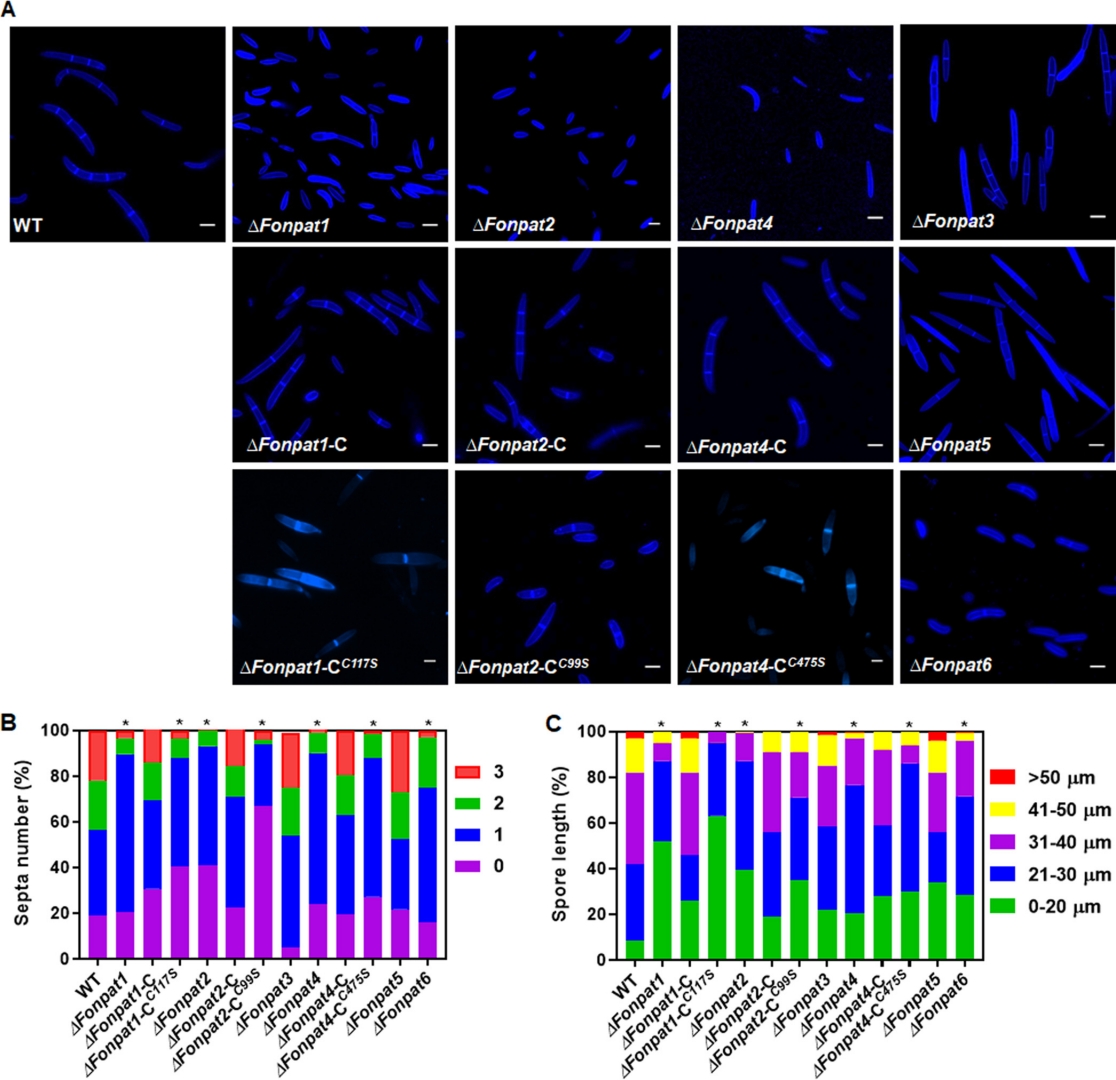
Functions of *FonPAT* genes in macroconidia morphology of *Fon*. (A) Macroconidia morphology; (B) macroconidial septum number; (C) macroconidial length of the WT, deletion mutants *ΔFonpat1*, *ΔFonpat2*, *ΔFonpat3*, *ΔFonpat4*, *ΔFonpat5*, and *ΔFonpat6*, and complementation strains expressing *FonPAT1*-C, *FonPAT2*-C, *FonPAT4*-C, or DHHC-mutated variants *FonPAT1*-C^C117S^, *FonPAT2*-C^C99S^, or *FonPAT4-*C^C475S^. Macroconidia produced by the indicated strains were stained with 10 μg/mL CFW and examined under a differential interference contrast microscope for their morphology, septum number, and length. (Scale bar in panel B) = 50 μm. Experiments were independently performed three times with similar results, and results from one representative experiment are shown in panel A. The data presented in panels B and C are the means ± SD from three independent experiments, and asterisks indicate the significant difference at *P < *0.05.

The functions of *FonPATs* in basic biological processes of *Fon* were investigated by analyzing the vegetative growth, conidiation, spore germination, and mycelial and macroconidial morphology of the *FonPAT* deletion mutants. Mycelial growth of all 6 *ΔFonpat* deletion mutants on potato dextrose agar (PDA) medium or minimal medium (MM) was significantly slower than that of the WT (Fig. 2A, C, and D). *ΔFonpat2* grew irregular curvature mycelia, while other deletion mutants grew normal mycelia compared with the WT (Fig. 2B). After 48 h of incubation in mung bean liquid (MBL) medium, *ΔFonpat1*, *ΔFonpat2*, *ΔFonpat3*, *ΔFonpat4*, and *ΔFonpat5* produced significantly fewer conidia, while *ΔFonpat6* generated a comparable number of conidia compared to the WT (Fig. 2E). Spore germination rates of all *FonPAT* deletion mutants except *ΔFonpat6* were significantly reduced compared with that of the WT (Fig. 2F). Macroconidia produced by *ΔFonpat1*, *ΔFonpat2*, *ΔFonpat4*, and *ΔFonpat6* differed morphologically from the WT in terms of size and septum number, while macroconidia generated by *ΔFonpat3* and *ΔFonpat5* were similar to those of the WT (Fig. 3A). The septum numbers of macroconidia from *ΔFonpat3* and *ΔFonpat5* were comparable to that of the WT, while the proportion of macroconidia with less than 1 septum was higher in *ΔFonpat1*, *ΔFonpat2*, *ΔFonpat4*, and *ΔFonpat6* than that of the WT (Fig. 3B). Furthermore, the macroconidia from all deletion mutants were shorter in length than the WT, and this was clearly evident for the macroconidia from *ΔFonpat1* and *ΔFonpat2* (Fig. 3C). The mycelial growth, conidiation, spore germination, and mycelial and conidial morphology of the complementation strains *ΔFonpat1*-C, *ΔFonpat2*-C, and *ΔFonpat4*-C were restored to those of the WT (Fig. 2C to E and Fig. 3A to C). These results suggest that *FonPAT* genes play critical roles in regulating various basic biological processes, e.g., vegetative growth and hyphal morphology, conidiation and conidial morphology, and spore germination, in *Fon*.

### Functions of *FonPAT* genes in the stress response of *Fon*.

The involvement of *FonPAT* genes in the environmental stress response of *Fon* was also studied by examining the *FonPAT* deletion mutants for their mycelial growth in response to exogenous stress factors such as Congo red (CR), calcofluor white (CFW), sodium dodecyl sulfate (SDS), H_2_O_2_, NaCl, CaCl_2_, and paraquat. Generally, the *FonPAT* deletion mutants showed distinct mycelial growth phenotypes in response to these stress factors. In particular, the *FonPAT* deletion mutants became more sensitive to SDS but more resistant to CR, CFW, paraquat, NaCl, and CaCl_2_ (Fig. S5A and B). Δ*Fonpat1*, Δ*Fonpat2*, Δ*Fonpat3*, and *ΔFonpat4* became less sensitive to H_2_O_2_, while *ΔFonpat5* and *ΔFonpat6* had similar H_2_O_2_ sensitivity to the WT (Fig. S5A and B). These results indicate that *FonPAT* genes play vital but distinct roles in response to cell wall-perturbing agents and oxidative stress and thus are involved in the stress response in *Fon*.

### FonPAT1, FonPAT2, and FonPAT4 are functional PATs.

**FIG 4:**
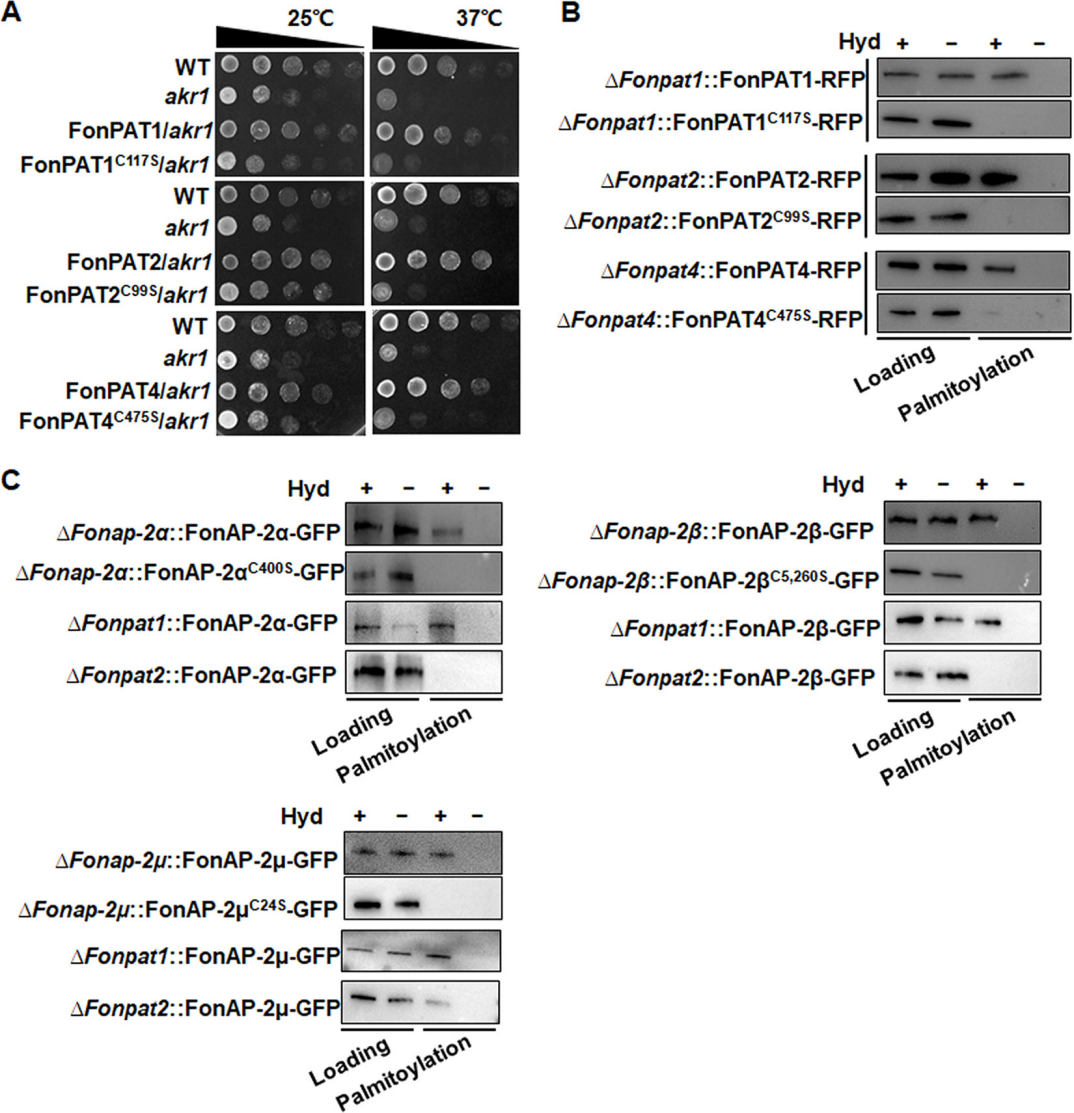
FonPAT1, FonPAT2, and FonPAT4 are functional PATs, and FonPAT2 palmitoylates the FonAP-2 complex subunits FonAP-2α, FonAP-2β, and FonAP-2μ. (A) Complementation of the thermosensitive defeat of yeast *akr1* by FonPAT1, FonPAT2 and FonPAT4. The WT and *ark1* transformed with pYES2 vector, pYES2-FonPAT1, pYES2-FonPAT1^C117S^, pYES2-FonPAT2, pYES2-FonPAT2^C99S^, pYES2-FonPAT4, or pYES2-FonPAT4^C475S^ were grown at 25°C or 37°C for 2 days and then incubated at 28°C for 1 day. PAT activity of FonPAT1, FonPAT2, and FonPAT4 was assessed by the growth performance of the yeasts at 37°C, with the growth performance at 25°C as the control. (B) FonPAT1, FonPAT2, and FonPAT4 were auto-palmitoylated in the ABE assay. (C) FonPAT2 but not FonPAT1 palmitoylated FonAP-2α, FonAP-2β, and FonAP-2μ. FonAP-2α-GFP, FonAP-2β-GFP, and FonAP-2μ-GFP and their palmitoylation-deficient variants FonAP-2α^C400S^-GFP, FonAP-2β^C5,260S^-GFP, and FonAP-2μ^C24S^-GFP were introduced into the indicated strains, and the palmitoylation level was detected using the ABE assay. Hyd, hydroxylamine. The loading lanes indicate equal amounts of proteins loaded to NeutrAvidin beads, while the palmitoylation lanes show the levels of proteins recovered from NeutrAvidin beads. Experiments were independently performed three times with similar results, and results from one representative experiment are shown.

The PAT activity of FonPAT1, FonPAT2, and FonPAT4 was investigated by complementation of yeast *akr1* and auto-palmitoylation assays. Considering the importance of the cysteine residue in DHHC motifs of the CRD domain in the enzymatic activity of PATs (19, 23), DHHC-mutated variants FonPAT1^C117S^, FonPAT2^C99S^, and FonPAT4^C475S^ were generated and examined for their activity. Yeast Akr1 has PAT activity, and the loss-of-function mutant *akr1* shows a temperature-sensitive growth defect, with normal growth occurring at 25°C but poor and aberrant growth occurring at 30°C (53, 54). All yeast strains transformed with each of the recombinant pYES2 vectors showed a growth phenotype similar to that of the WT and the *akr1* mutant at 25°C (Fig. 4A). When grown on a galactose minimal plate at 37°C, the *akr1* mutant did not grow; in contrast, the *akr1* mutant transformed with pYES2-FonPAT1, pYES2-FonPAT2, or pYES2-FonPAT4 did grow (Fig. 4A). However, the *akr1* mutants transformed with pYES2-FonPAT1^C117S^, pYES2-FonPAT2^C99S^, or pYES2-FonPAT4^C475S^ failed to grow on galactose minimal plates at 37°C (Fig. 4A). The rescue of the temperature-sensitive defect of yeast *akr1* at 37°C indicates that FonPAT1, FonPAT2, and FonPAT4 have PAT activity and that the cysteine residue in DHHC motifs is critical to the enzymatic activity of FonPAT1, FonPAT2, and FonPAT4.

The acyl-biotin exchange (ABE) assay, commonly used to examine whether a protein is palmitoylated (55), was performed to further confirm the PAT activity of FonPAT1, FonPAT2, and FonPAT4 and determine whether they were auto-palmitoylated. For this purpose, FonPAT1-RFP, FonPAT2-RFP, and FonPAT4-RFP and the DHHC-mutated variants FonPAT1^C117S^-RFP, FonPAT2^C99S^-RFP, and FonPAT4^C475S^-RFP were transferred into the corresponding deletion mutants. The unmodified cysteine thiol groups on FonPAT1-RFP, FonPAT1^C117S^-RFP, FonPAT2-RFP, FonPAT2^C99S^-RFP, FonPAT4-RFP, and FonPAT4^C475S^-RFP in the fungal total protein extracts were first blocked by the sulfhydryl reactive reagent *N*-ethylmaleimide (NEM) and then treated with the *S*-acyl group cleavage reagent hydroxylamine, which released the thioester-linked palmitoyl moieties and restored the modified cysteine to thiols. The proteins unmodified cysteine thiol groups were biotinylated with a thiol-reactive biotinylation reagent *N*-[6-(biotinamido)hexyl]-3’-(2’-pyridyldithio)-propionamide (biotin-HPDP), immobilized onto neutravidin agarose beads, and detected by Western blotting. Under equal loading condition, clear bands corresponding to FonPAT1-RFP, FonPAT2-RFP, and FonPAT4-RFP were detected in protein samples extracted with hydroxylamine from *ΔFonpat1*::*FonPAT1-RFP*, *ΔFonpat2*::*FonPAT2-RFP*, and *ΔFonpat4*::*FonPAT4-RFP* strains, while no band was observed in the protein samples extracted without hydroxylamine (Fig. 4B). However, no bands corresponding to FonPAT1-RFP, FonPAT2-RFP, and FonPAT4-RFP were detected in protein samples extracted with or without hydroxylamine from *ΔFonpat1*::*FonPAT1*^C117S^*-RFP*, *ΔFonpat2*::*FonPAT2*^C99S^*-RFP*, and *ΔFonpat4*::*FonPAT4*^C475S^*-RFP* strains (Fig. 4B). These results demonstrated that FonPAT1, FonPAT2, and FonPAT4 were auto-palmitoylated *in vivo* and further confirmed that they are functional PATs in *Fon*.

### The DHHC motifs are critical to the functions of FonPAT1, FonPAT2, and FonPAT4.

The significance of the DHHC motifs in FonPAT1, FonPAT2, and FonPAT4 was further investigated by analyzing the phenotypes of the *ΔFonpat1*::*FonPAT1*^C117S^*-RFP*, *ΔFonpat2*::*FonPAT2*^C99S^*-RFP*, and *ΔFonpat4*::*FonPAT4*^C475S^*-RFP* strains in basic biological processes and virulence and stress responses. Pathogenicity toward watermelon (Fig. 1C to G), vegetative growth and hyphal morphology (Fig. 2 and 3), conidiation and conidial morphology (Fig. 2 and 3), responses to exogenous stress factors (e.g., CR, CFW, SDS, H_2_O_2_, NaCl, CaCl_2_, and paraquat) (Fig. S5), and penetration ability (see Fig. S4B) of the *ΔFonpat1*::*FonPAT1*^C117S^*-RFP*, *ΔFonpat2*::*FonPAT2*^C99S^*-RFP*, and *ΔFonpat4*::*FonPAT4*^C475S^*-RFP* strains were not completely restored to the levels in the WT and their corresponding complementation strains *ΔFonpat1*-C, *ΔFonpat2*-C, and *ΔFonpat4*-C but were similar to those in the deletion mutants *ΔFonpat1*, *ΔFonpat2*, and *ΔFonpat4*. Subcellular localization assays showed that FonPAT1-RFP, FonPAT2-RFP, FonPAT1^C117S^-RFP, and FonPAT2^C99S^-RFP were localized to the endoplasmic reticulum (ER), colocalized with the ER marker (see Fig. S6). Together, these results indicate that the DHHC motifs are critical for the functions of FonPAT1, FonPAT2, and FonPAT4 in regulating the basic biological processes and virulence and stress responses of *Fon*, but are not involved in regulating the subcellular localization of FonPAT1 and FonPAT2.

### Characterization of FonPAT2-palmitoylated substrates.

To explore the molecular mechanism of FonPAT2 in *Fon* virulence, the ABE assay coupled with a liquid chromatography-mass spectrometry (LC-MS) approach was performed to characterize the FonPAT2-palmitoylated substrates by comparatively analyzing the palmitoylated proteins present in the WT but absent in *ΔFonpat2* (33). The ABE/LC-MS assay identified a total of 211 putative palmitoylated proteins that were present in the WT but absent in *ΔFonpat2* in 2 independent biological replicates, and 36 palmitoylated proteins overlapped in the two replicates (Table S1). Some of the putative palmitoylated proteins were previously reported to be involved in the pathogenicity of filamentous fungi such as F. graminearum, Botrytis cinerea, Verticillium dahliae, and Aspergillus flavus, for example, FOXG_00780 (AP-2 complex subunit α) and FOXG_04448 (AP-2 complex subunit μ) (41, 42), FOXG_05141 (autophagy-related ATG7) and FOXG_01866 (ATG20) (56–58), FOXG_11305 (myosin-1) (59), FOXG_19023 (chitin synthase) (60), FOXG_13911 (Ras-like protein Rab-11B), and FOXG_03575 (Ras-like C3 botulinum toxin substrate 1) (61), and FOXG_11168 (ankyrin repeat protein NUC-2) (62). Meanwhile, homologues for some of the putative palmitoylated proteins were previously shown to be palmitoylated in yeast and other organisms, for example, FOXG_08214 (synaptobrevin-like YKT6) (63), FOXG_04579 (actin γ) (33), FOXG_13911 (Ras-like protein Rab-11B), FOXG_03575 (Ras-like C3 botulinum toxin substrate 1) (64), and FOXG_00122 (Ca^2+^ transporting ATPase) (33). These data indicate that FonPAT2 can palmitoylate a group of proteins in *Fon*, some of which are related to pathogenicity, further demonstrating the importance of FonPAT2 in *Fon* virulence. Palmitoylation of proteins usually happens at specific cysteine residues (19); however, no sequence conservation or preference flanking the palmitoylation site in the FonPAT2-palmitoylated substrates was observed.

### Palmitoylation of FonAP-2 complex subunits by FonPAT2.

A previous study has shown that the FgAP-2 complex is essential for virulence in F. graminearum (41). The appearance of the FonAP-2 complex subunits α and μ as the FonPAT2-palmitoylated proteins implied that the FonAP-2 complex subunits are the FonPAT2 substrates whose function may depend on the FonPAT2-mediated palmitoylation in *Fon*. To test this hypothesis, the FonAP-2 complex subunits were chosen for further study. In yeast and F. graminearum, the AP-2 complex consists of four subunits, including two large subunits, AP-2α and AP-2β, a core subunit, AP-2μ, and a small subunit, AP-2σ (37, 41). A blastp search identified four subunits in the F. oxysporum f. sp. *lycopersici* genome, which were cloned from *Fon* and named FonAP-2α (FOXG_00780), FonAP-2β (FOXG_08330), FonAP-2μ (FOXG_04448), and FonAP-2σ (FOXG_08592) (see Fig. S7A). Phylogenetic tree analysis showed that the FonAP-2 complex subunits were highly related to their counterparts from different fungi, including yeast, F. graminearum, and M. oryzae (see Fig. S7B). ABE/LC-MS analysis revealed a single palmitoylation site at cysteine 400 in FonAP-2α and at cysteine-24 in FonAP-2μ (see Fig. S7A; Table S1), and these sites were confirmed by the prediction using the CSS-Palm 4.0 algorithm. Furthermore, the CSS-Palm 4.0 algorithm also identified two palmitoylation sites at cysteines 5 and 260 in FonAP-2β; however, no putative palmitoylation site was predicted in FonAP-2σ (see Fig. S7A). These data imply that the FonAP-2 complex subunits α, β, and μ may be palmitoylated by FonPAT2.

Targeted deletion mutants *ΔFonap-2α*, *ΔFonap-2β*, *ΔFonap-2μ*, and *ΔFonap-2σ* were generated by the homologous recombination strategy (see Fig. S8A). Fragments with different sizes in the WT and the corresponding deletion mutants were detected in PCR with pairs of primers located in the regions flanking the deletion targets in the *FonAP-2* genes (see Fig. S8B). A single hybridizing band was detected in each of the deletion mutants, but not in the WT, when hybridized with the *HPH* probe in Southern blotting (see Fig. S8C). The transcript levels of each of the FonAP-2 complex subunit genes were undetectable in *ΔFonap-2μ* and less than 4% in *ΔFonap-2α*, *ΔFonap-2β*, and *ΔFonap-2σ* (see Fig. S8D). Meanwhile, complementation strains were also generated by separately introducing FonAP-2α-GFP, FonAP-2β-GFP, and FonAP-2μ-GFP and their palmitoylation-deficient variants FonAP-2α^C400S^-GFP, FonAP-2β^C5,260S^-GFP, and FonAP-2μ^C24S^-GFP into *ΔFonap-2α*, *ΔFonap-2β*, and *ΔFonap-2μ*, respectively, to assess the *in vivo* palmitoylation of FonAP-2α, FonAP-2β, and FonAP-2μ. Under equal loading conditions, clear palmitoylated FonAP-2α-GFP, FonAP-2β-GFP, and FonAP-2μ-GFP bands were detected in protein samples extracted with hydroxylamine from *ΔFonap-2α*::*FonAP-2α-GFP*, *ΔFonap-2β*::*FonAP-2β-GFP*, and *ΔFonap-2μ*::*FonAP-2μ-GFP* strains, respectively, while no band was observed in protein samples extracted without hydroxylamine (Fig. 4C). However, no palmitoylated FonAP-2α^C400S^-GFP, FonAP-2β^C5,260S^-GFP, or FonAP-2μ^C24S^-GFP bands were detected in protein samples extracted with or without hydroxylamine from *ΔFonap-2α*::*FonAP-2α*^C400S^*-GFP*, *ΔFonap-2β*::*FonAP-2β*^C5,260S^*-GFP*, and *ΔFonap-2μ*::*FonAP-2μ*^C24S^*-GFP* strains (Fig. 4C). These results demonstrated that the FonAP-2 complex subunits FonAP-2α, FonAP-2β, and FonAP-2μ were palmitoylated *in vivo* at C400, C5/C260, and C24, respectively.

To examine whether the palmitoylation of FonAP-2α, FonAP-2β, and FonAP-2μ was specifically catalyzed by FonPAT2, the *in vivo* palmitoylation of FonAP-2α-GFP, FonAP-2β-GFP, and FonAP-2μ-GFP was assessed in *ΔFonpat1* or *ΔFonpat2*. Under equal loading conditions, palmitoylated FonAP-2α-GFP, FonAP-2β-GFP, and FonAP-2μ-GFP bands were detected in *ΔFonpat1* (Fig. 4C). No significant palmitoylated FonAP-2α-GFP or FonAP-2β-GFP bands were observed, while a weaker palmitoylated FonAP-2μ-GFP band was visible in *ΔFonpat2* (Fig. 4C). These results indicated that the palmitoylation of FonAP-2α, FonAP-2β, and FonAP-2μ was specifically catalyzed by FonPAT2 but not by FonPAT1 and that the palmitoylation of FonAP-2μ may also be catalyzed by other unknown FonPATs.

### The FonAP-2 complex subunits and their palmitoylation are critical to the function in *Fon* virulence.

**FIG 5:**
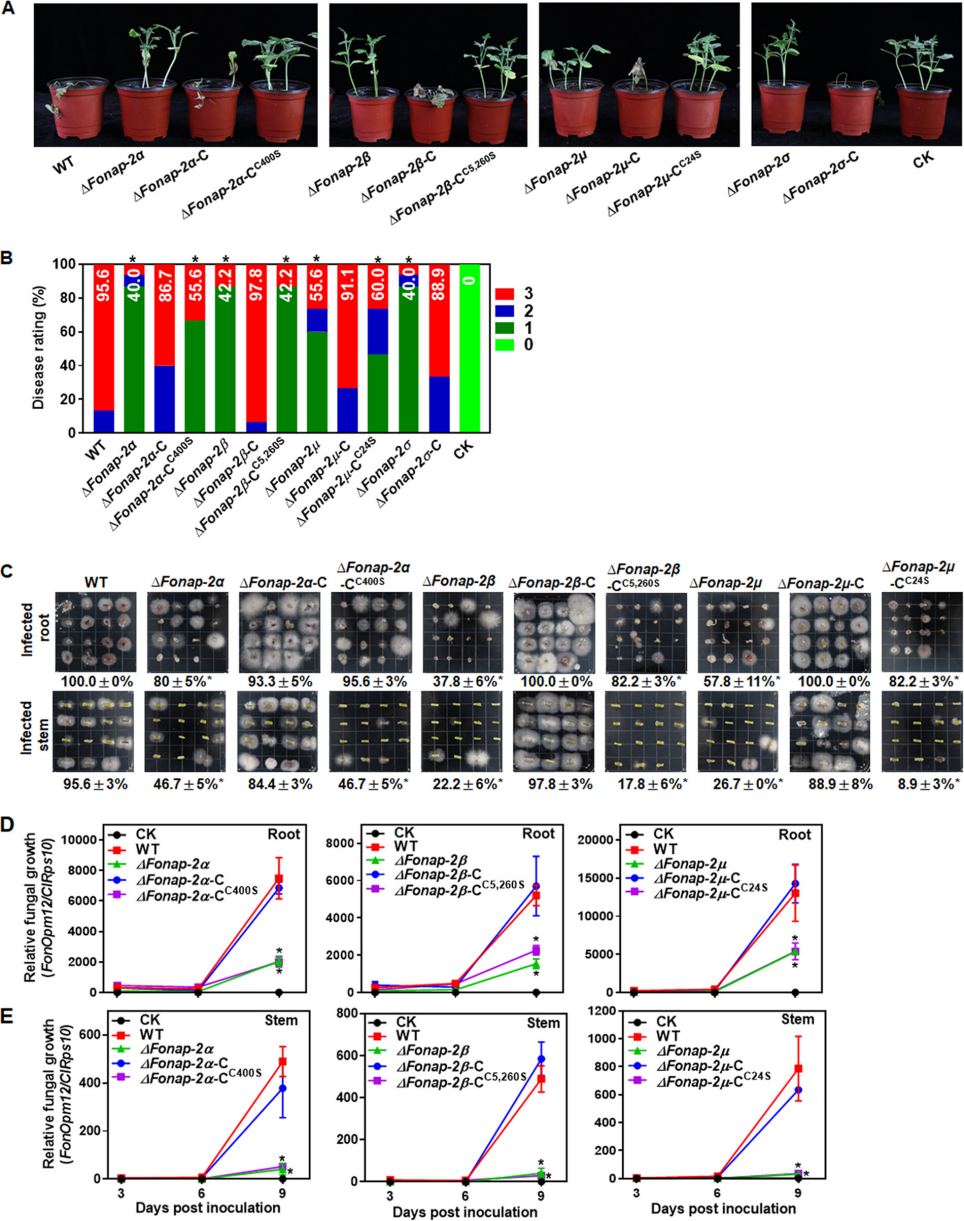
FonAP-2 complex subunits and their palmitoylation are required for *Fon* virulence on watermelon. (A) Disease phenotype of plants inoculated with the WT, deletion mutants *ΔFonap-2α*, *ΔFonap-2β*, *ΔFonap-2μ*, and *ΔFonap-2σ*, and complementation strains expressing FonAP-2α, FonAP-2α^C400S^, FonAP-2β, FonAP-2β^C5,260S^, FonAP-2μ, FonAP-2μ^C24S^, or FonAP2σ. CK, plants inoculated with a preparation of mung bean broth without *Fon* spores. (B) Disease ratings of plants inoculated with the WT, deletion mutants, and complementation strains at 21 dpi. (C) Fungal colonies recovered from roots and stems of plants inoculated with WT, *ΔFonap-2α*, *ΔFonap-2β*, *ΔFonap-2μ*, and their complementation strains. (D and E) *In planta* fungal growth in roots and stems of plants inoculated with WT, *ΔFonap-2α*, *ΔFonap-2β*, *ΔFonap-2μ*, and complementation strains expressing FonAP-2α, FonAP-2α^C400S^, FonAP-2β, FonAP-2β^C5,260S^, FonAP-2μ, or FonAP-2μ^C24S^. Experiments were independently performed three times with similar results, and results from one representative experiment are shown in panels A and C. The data presented in panels B, D, and E are the means ± SD from three independent experiments; asterisks indicate the significant difference at *P < *0.05.

In repeated experiments, the *ΔFonap-2α*-, *ΔFonap-2β*-, *ΔFonap-2μ*-, and *ΔFonap-2σ*-inoculated plants showed fewer disease symptoms and reduced disease severity, leading to reductions of disease severity of 58.2%, 55.9%, 41.8%, and 58.2%, respectively, compared with that in WT-inoculated plants (Fig. 5A and B). In contrast, the complementation strain *ΔFonap-2α*-C-, *ΔFonap-2β*-C-, *ΔFonap-2μ*-C-, and *ΔFonap-2σ*-C-inoculated plants had comparable disease symptoms and severity to that in WT-inoculated plants (Fig. 5A and B). However, the palmitoylation-deficient variant-complemented strain *ΔFonap-2α*-C^C400S^-, *ΔFonap-2β*-C^C5,260S^-, and *ΔFonap-2μ*-C^C24S^-inoculated plants showed comparable disease symptoms and severity to those in their corresponding deletion mutants (Fig. 5A and B). Tissue-based examination of *in planta* fungal growth assays revealed that the percentages of root and stem segments supporting *Fon* growth and colony-forming from the *ΔFonap-2α*-, *ΔFonap-2β*-, and *ΔFonap-2μ*-inoculated plants and from the palmitoylation-deficient variant-complemented strain *ΔFonap-2α*-C^C400S^-, *ΔFonap-2β*-C^C5,260S^-, and *ΔFonap-2μ*-C^C24S^-inoculated plants were significantly decreased compared with those in the WT-inoculated plants (Fig. 5C). In contrast, the percentages from the complementation strain *ΔFonap-2α*-C-, *ΔFonap-2β*-C-, and *ΔFonap-2μ*-C-inoculated plants were comparable to those in the WT-inoculated plants (Fig. 5C). Similarly, relative fungal growth in roots and stems of the *ΔFonap-2α*-, *ΔFonap-2β*-, and *ΔFonap-2μ*-inoculated plants and the palmitoylation-deficient variant-complemented strain *ΔFonap-2α*-C^C400S^-, *ΔFonap-2β*-C^C5,260S^-, and *ΔFonap-2μ*-C^C24S^-inoculated plants were significantly decreased by 59% to 73%, and by 89% to 96% in stems, respectively, compared with those in WT-inoculated plants at 9 dpi (Fig. 5D and E). In contrast, no difference in the relative fungal growth in roots and stems was observed among the complementation strain *ΔFonap-2α*-C-, *ΔFonap-2β*-C-, and *ΔFonap-2μ*-C-inoculated plants and the WT-inoculated plants at 9 dpi (Fig. 5D and E). These results suggest that the FonAP-2 complex subunits are required for *Fon* virulence and that palmitoylation of FonAP-2α, FonAP-2β, and FonAP-2μ is critical to the function of the FonAP-2 complex in *Fon* virulence.

### The FonAP-2 complex subunits and their palmitoylation are critical to the functions in basic biological processes of *Fon*.

**FIG 6:**
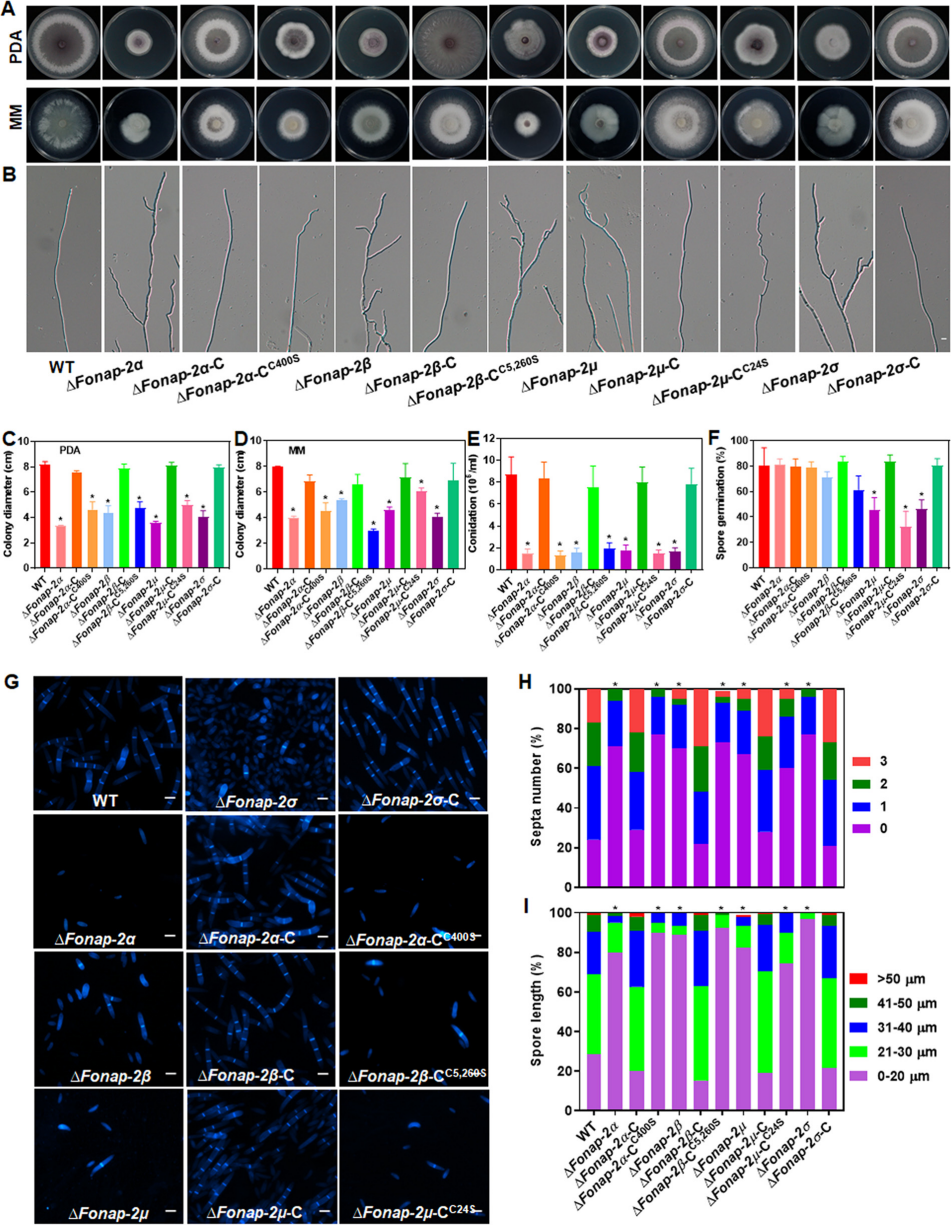
The FonAP-2 complex subunits and their palmitoylation are involved in the regulation of basic biological processes in *Fon*. (A to I) Growth phenotype (A), hyphal polarized growth (B), colony diameters on PDA (C) and MM (D) plates, conidiation (E), spore germination (F), macroconidia morphology (G), macroconidial septum number (H), and macroconidial length (I) of the WT, deletion mutants *ΔFonap-2α*, *ΔFonap-2β*, *ΔFonap-2μ*, and *ΔFonap-2σ*, and their complementation strains expressing *FonAP-2α*-C, *FonAP-2β*-C, *FonAP-2μ*-C, *FonAP-2σ*-C, or palmitoylation-deficient variants *FonAP-2α*-C^C400S^, *FonAP-2β*-C^C5,260S^, and *FonAP-2μ*-C^C24S^. The indicated strains were grown on PDA or MM plates for 7 days at 26°C to estimate the mycelial growth and observe the hyphal morphology under a differential interference contrast microscope or were grown in MBL broth for 2 days at 26°C to count the macroconidia. Macroconidia produced by the indicated strains were stained with 10 μg/mL CFW and examined under a differential interference contrast microscope for the morphology, septum number, and length. Macroconidia were suspended in YEPD broth and incubated for 12 h to estimate the germination status by examining at least 100 macroconidia. Experiments were independently performed three times with similar results, and results from one representative experiment are shown in panels A, B, and G. The data presented in panels (C to F, H, and I) are the means ± SD from three independent experiments; asterisks indicate the significant difference at *P < *0.05.

The possible function of FonAP-2 complex subunits and the significance of their palmitoylation in basic biological processes of *Fon* were investigated. Mycelial growth of *ΔFonap-2α*, *ΔFonap-2β*, *ΔFonap-2μ*, and *ΔFonap-2σ* on PDA or MM was significantly slower than that of the WT (Fig. 6A, C, and D). *ΔFonap-2α*, *ΔFonap-2β*, *ΔFonap-2μ*, and *ΔFonap-2σ* showed defects in normally polarized mycelial growth and grew irregular branched mycelia in comparison to the normally polarized unbranched mycelia in the WT (Fig. 6B). After 48 h of incubation in MBL, *ΔFonap-2α*, *ΔFonap-2β*, *ΔFonap-2μ*, and *ΔFonap-2σ* produced significantly fewer macroconidia, and germination rates of macroconidia from these deletion mutants were significantly reduced compared to those of the WT (Fig. 6E and F). Macroconidia produced by *ΔFonap-2α*, *ΔFonap-2β*, *ΔFonap-2μ*, and *ΔFonap-2σ* morphologically differed from those of the WT in terms of septum number and length (Fig. 6G); for example, >90% of macroconidia from these deletion mutants had less than 1 septum and were <30 μm long, compared with those from the WT (60% of macroconidia with less than 1 septum and 70% of them <30 μm long) (Fig. 6H and I). Furthermore, *ΔFonap-2α*, *ΔFonap-2β*, *ΔFonap-2μ*, and *ΔFonap-2σ* lost the penetration ability against cellophane membrane (see Fig. S8E). In these experiments, the complementation strains *ΔFonap-2α*-C, *ΔFonap-2β*-C, *ΔFonap-2μ*-C, and *ΔFonap-2σ*-C restored the above-mentioned features to the WT; however, the palmitoylation-deficient variant-complemented strains *ΔFonap-2α*-C^C400S^, *ΔFonap-2β*-C^C5,260S^, and *ΔFonap-2μ*-C^C24S^ showed similar phenotypes to the deletion mutants (Fig. 6A to I; see also Fig. S8E). Together, these results indicate that the FonAP-2 complex and the palmitoylation of FonAP-2α, FonAP-2β, and FonAP-2μ play critical roles in basic biological processes, e.g., vegetative growth and mycelial morphology, conidiation and conidial morphology, spore germination, and penetration ability, in *Fon*.

### The FonAP-2 complex subunits and their palmitoylation are critical to the cell wall integrity of *Fon*.

**FIG 7:**
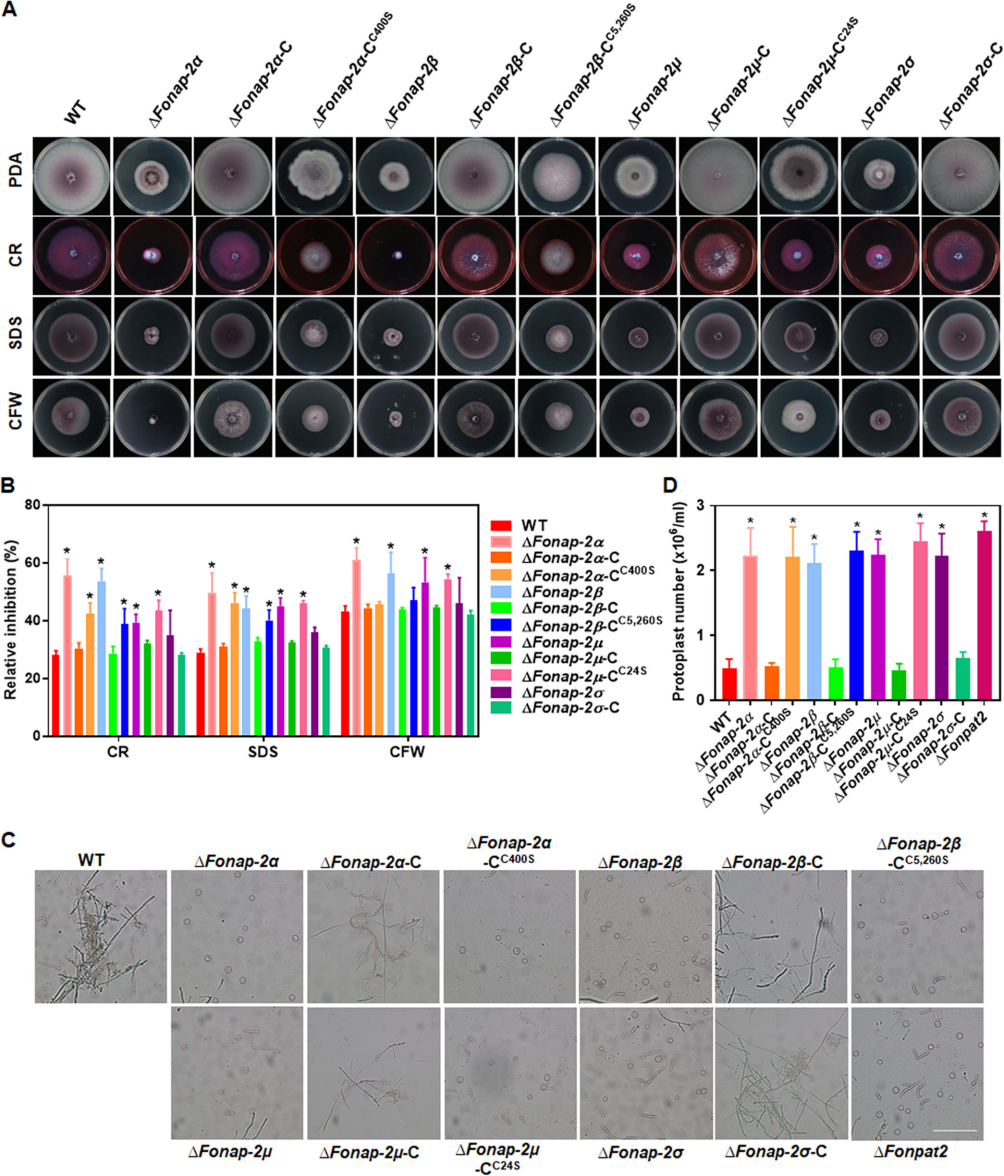
Palmitoylation of the FonAP-2 complex subunits is involved in the cell wall integrity of *Fon*. (A and B) Growth phenotype (A) and inhibition rates of the mycelial growth (B) of the WT, deletion mutants *ΔFonap-2α*, *ΔFonap-2β*, *ΔFonap-2μ*, and *ΔFonap-2σ*, and their complement strains expressing *FonAP-2α*-C, *FonAP-2β*-C, *FonAP-2μ*-C, and *FonAP-2σ*-C or palmitoylation-deficient variants *FonAP-2α-*C^C400S^, *FonAP-2β-*C^C5,260S^, and *FonAP-2μ-*C^C24S^ grown on PDA plates supplemented with 0.2 g/L CFW, 0.2 g/L CR, or 0.3 g/L SDS. (C) Sensitivity to digestion by cell wall-degrading enzymes; (D) numbers of protoplasts released after treatment. Mycelia of the indicated strains were treated for 2 h at 30°C in a solution containing the same amounts of lysozyme, cellulase, and driselase, and the released protoplasts were microscopically examined and photographed. Bar = 20 μm. Experiments were independently performed three times with similar results, and results from one representative experiment are shown in panels A and C. The data presented in panels B and D are the means ±SD from three independent experiments; and asterisks indicate the significant difference at *P < *0.05.

The involvement of FonAP-2 complex subunits and the significance of their palmitoylation to cell wall integrity of *Fon* were also explored. In cell wall stress assays, the inhibition of mycelial growth of *ΔFonap-2α*, *ΔFonap-2β*, and *ΔFonap-2μ* by CFW, CR, or SDS was significantly increased compared to that of the WT (Fig. 7A and B). However, the growth inhibition of *ΔFonap-2σ* by CFW, CR, or SDS was comparable to that of the WT (Fig. 7A and B). In cell wall-degrading sensitivity assays, the *ΔFonap-2α*, *ΔFonap-2β*, *ΔFonap-2μ*, and *ΔFonap-2σ* mycelia showed increased sensitivity to the cell wall degradation treatment by a combination of cellulase, driselase, and lysozyme (Fig. 7C), leading to significant increases of protoplast release by 4- to 5-fold (Fig. 7D). Additionally, *ΔFonpat2* also showed an increased sensitivity to the cell wall degradation treatment (Fig. 7C and D). The complementation strains *ΔFonap-2α*-C, *ΔFonap-2β*-C, *ΔFonap-2μ*-C, and *ΔFonap-2σ*-C restored the cell wall stress and degradation sensitivities to the WT (Fig. 7A to D). The palmitoylation-deficient variant-complemented strains *ΔFonap-2α*-C^C400S^, *ΔFonap-2β*-C^C5,260S^, and *ΔFonap-2μ*-C^C24S^ exhibited similar cell wall stress and degradation sensitivities to those in the deletion mutants (Fig. 7A to D). Collectively, these results indicate that the FonAP-2 complex and the palmitoylation of FonAP-2α, FonAP-2β, and FonAP-2μ are critical for the cell wall integrity in *Fon*.

### Palmitoylation of FonAP-2α, FonAP-2β, and FonAP-2μ is essential for the formation and stability of the FonAP-2 complex in *Fon*.

**FIG 8:**
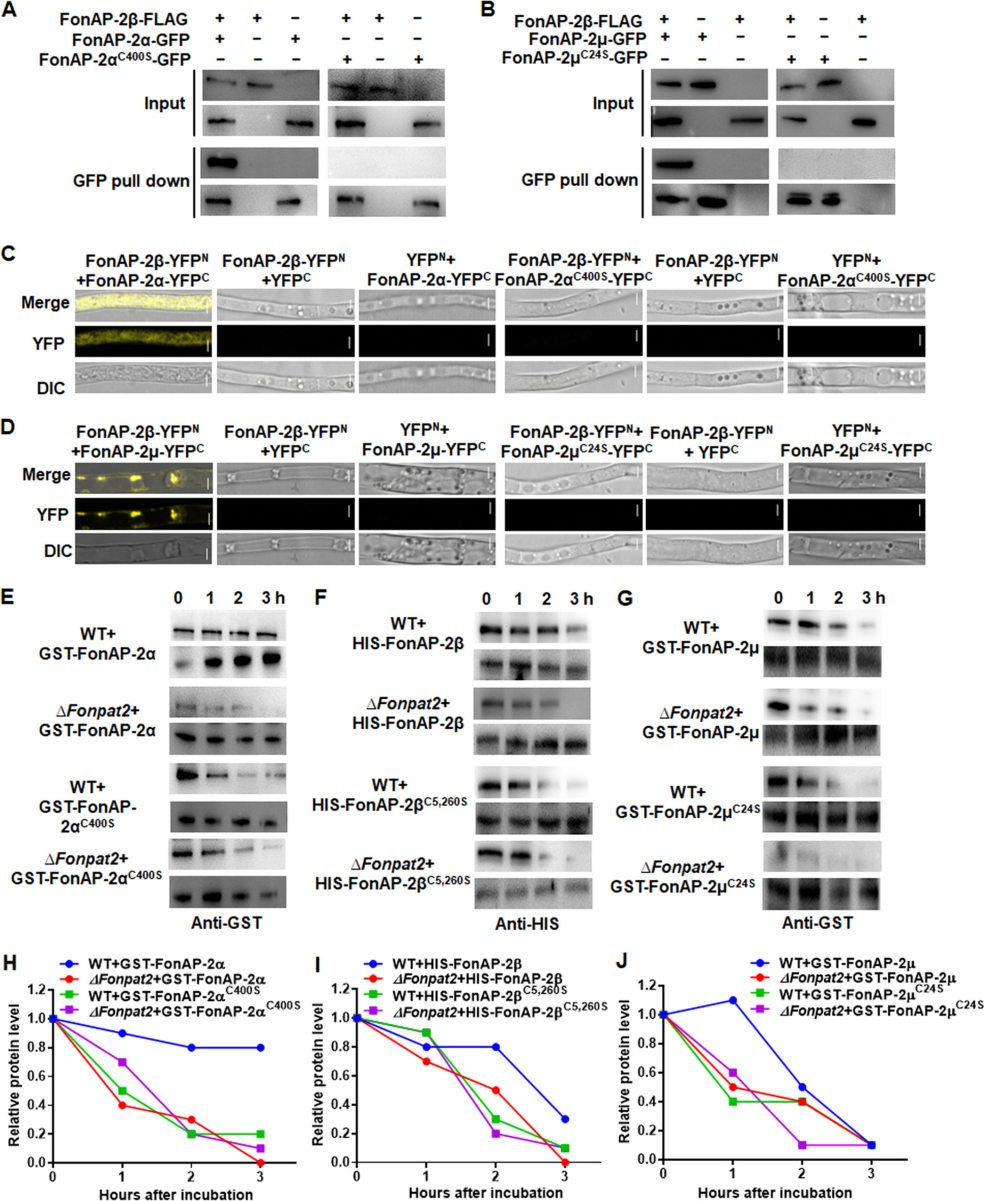
Palmitoylation is critical to the formation and stability of the FonAP-2 complex in *Fon*. (A and B) Interactions between FonAP-2β and FonAP-2α or FonAP-2α^C400S^ (A) and between FonAP-2β and FonAP-2μ or FonAP-2μ^C24S^ (B) in co-IP assays. Proteins were extracted from strains coexpressing FonAP-2β-FLAG/FonAP-2α-GFP, FonAP-2β-FLAG/FonAP-2α^C400S^-GFP, FonAP-2β-FLAG/FonAP-2μ-GFP, or FonAP-2β-FLAG/FonAP-2μ^C24S^-GFP and immunoprecipitated with anti-GFP agarose beads, followed by detection using anti-GFP and anti-FLAG antibodies, respectively. (C and D) Interactions between FonAP-2β and FonAP-2α or FonAP-2α^C400S^ (C) and between FonAP-2β and FonAP-2μ or FonAP-2μ^C24S^ (D) in BiFC assays. Bar = 2.5 μm. (E to G) Stability of FonAP-2α and FonAP-2α^C400S^ (E), FonAP-2β and FonAP-2β^C5,260S^ (F), and FonAP-2μ and FonAP-2μ^C24S^ (G) in *in vitro* cell-free degradation assays. (H to J) Degradation dynamics of FonAP-2α and FonAP-2α^C400S^ (H), FonAP-2β and FonAP-2β^C5,260S^ (I), and FonAP-2μ and FonAP-2μ^C24S^ (J) in *in vitro* cell-free degradation assays. Recombinant GST-FonAP-2α, GST-FonAP-2α^C400S^, HIS-FonAP-2β, HIS-FonAP-2β-C^C5,260S^, GST-FonAP-2μ, and GST-FonAP-2μ^C24S^ were coincubated with total proteins extracted from the WT and *ΔFonpat2*, and the protein levels were detected by Western blotting using anti-GST or anti-HIS antibodies. Relative levels of the target bands were quantified by ImageJ software. GAPDH was used as a loading control. Experiments were independently performed three times with similar results, and results from one representative experiment are shown.

The interactions among the FonAP-2 complex subunits FonAP-2α, FonAP-2β, and FonAP-2μ were examined. In coimmunoprecipitation (co-IP) and bimolecular fluorescence complementation (BiFC) assays, FonAP-2β interacted with FonAP-2α and FonAP-2μ, while FonAP-2β was unable to interact with the palmitoylation-deficient FonAP-2α^C400S^ and FonAP-2μ^C24S^ (Fig. 8A to D). These results demonstrated that palmitoylation of FonAP-2α, FonAP-2β, and FonAP-2μ is essential for the FonAP-2β-FonAP-2α and FonAP-2β-FonAP-2μ interactions, ensuring formation of the FonAP-2 complex in *Fon*.

The effect of palmitoylation on the stability of FonAP-2α, FonAP-2β, and FonAP-2μ was also studied using cell-free degradation assays. GST-FonAP-2α, HIS-FonAP-2β, and GST-FonAP-2μ were more stable incubated with total proteins extracted from the WT strain than those incubated with total proteins extracted from *ΔFonpat2* (Fig. 8E to J). In contrast, the degradation dynamics of GST-FonAP-2α^C400S^, HIS-FonAP-2β^C5,260S^, and GST-FonAP-2μ^C24S^ were comparable in the presence of total proteins extracted from the WT and *ΔFonpat2* and similar to the dynamics of GST-FonAP-2α, HIS-FonAP-2β, and GST-FonAP-2μ incubated with total proteins extracted from *ΔFonpat2* (Fig. 8E to J). Importantly, the degradation of GST-FonAP-2α^C400S^, HIS-FonAP-2β^C5,260S^, and GST-FonAP-2μ^C24S^ was faster than that of GST-FonAP-2α, HIS-FonAP-2β, and GST-FonAP-2μ, in the presence of total proteins extracted from the WT (Fig. 8E to J). These data indicate that palmitoylation of FonAP-2α, FonAP-2β, and FonAP-2μ favors their stability and thus contributes to the formation the FonAP-2 complex in *Fon*.

### Palmitoylation of FonAP-2α, FonAP-2β, and FonAP-2μ does not affect the subcellular localization in *Fon*.

The effect of palmitoylation on the subcellular localization of FonAP-2α, FonAP-2β, and FonAP-2μ was examined. mCherry fluorescent signal from FonAP-2α-mCherry and FonAP-2α^C400S^-mCherry in *ΔFonap-2α*, FonAP-2β-mCherry and FonAP-2β^C5,260S^-mCherry in *ΔFonap-2β*, and FonAP-2μ-mCherry and FonAP-2μ^C24S^-mCherry in *ΔFonap-2μ* was colocalized with the green fluorescent protein (GFP) fluorescent signal from the mito-tracker (see Fig. S9). Furthermore, the mCherry fluorescent signal from FonAP-2α-mCherry, FonAP-2β-mCherry, and FonAP-2μ-mCherry in *ΔFonpat2* also overlapped the GFP fluorescent signal from the mito-tracker (see Fig. S9). These observations indicate that the FonAP-2 complex normally localizes in mitochondria and that palmitoylation of FonAP-2α, FonAP-2β, and FonAP-2μ does not affect the subcellular localization of the FonAP-2 complex in *Fon*.

## DISCUSSION

The functions of PATs have been studied extensively in plants and yeasts (17–21); however, the roles of PATs and palmitoylation have been barely studied in filamentous fungi, especially in plant-pathogenic fungi. The present study demonstrated that FonPAT1, FonPAT2, and FonPAT4 are required for virulence in *Fon* and illustrated that FonPAT2 palmitoylates the FonAP-2 complex subunits FonAP-2α, FonAP-2β, and FonAP-2μ, whose palmitoylation is essential for the function of the FonAP-2 complex in *Fon* virulence. These findings not only provide novel insights into the molecular regulatory mechanism of virulence in *Fon*, but also shed a light on the importance of protein palmitoylation in virulence in plant-pathogenic fungi.

The FonPATs, including FonPAT1, FonPAT2, and FonPAT4, contain the characteristic DHHC motifs in the CRD domains. Data from the yeast *akr1* complementation and ABE assays demonstrate that FonPAT1, FonPAT2, and FonPAT4 are indeed functional PATs with auto-palmitoylation activity in *Fon*. This is consistent with the previous observation that some PATs are auto-palmitoylated *in vivo* (50, 65). The DHHC motifs in the CRD domain are generally necessary for enzymatic activity of PATs (19, 23), although the DHHC motifs seem to be not absolutely required for activity of yeast ScSwf1 and ScPfa4 (66). The failure of the FonPAT1^C117S^, FonPAT2^C99S^, and FonPAT4^C475S^ variants to restore the temperature-sensitive growth defect of the yeast *akr1* mutant and to be auto-palmitoylated clearly demonstrates that the DHHC motifs are essential for PAT activity in FonPAT1, FonPAT2, and FonPAT4. Importantly, FonPAT1^C117S^, FonPAT2^C99S^, and FonPAT4^C475S^ were also unable to rescue the defects in vegetative growth, asexual reproduction, stress response, and virulence of the *ΔFonpat1*, *ΔFonpat2*, and *ΔFonpat4* mutants. Collectively, these data confirm that FonPAT1, FonPAT2, and FonPAT4 function as PATs through their enzymatic catalytic DHHC motifs in the CRD domains and that the DHHC motif-conferred PAT activity is critical for the biological functions of FonPAT1, FonPAT2, and FonPAT4 in *Fon*.

The functions of PATs in fungal growth and development have been well documented. For example, some PATs are involved in vegetative growth and asexual reproduction in filamentous fungi such as A. nidulans and A. fumigatus (32, 33). The *FonPAT* deletion mutants exhibited defects in mycelial growth, conidiation, conidial morphology, and spore germination. In particular, *ΔFonpat2* mycelia showed an irregular curved growth phenotype, which was not observed in other *FonPAT* deletion mutants, indicating a special role for FonPAT2 in maintaining the polarized growth of the mycelial tip. The yeast ScSwf1, closely related to FonPAT2, is also involved in regulation of the actin cytoskeleton and cell polarized growth (66), implying a conserved function for the phylogenetically related FonPAT2 and ScSwf1. On the other hand, PATs have been implicated in the response of A. nidulans and C. neoformans to cell wall and metal ion stress (30, 33). Deletion of *FonPAT* genes significantly affected the sensitivity to cell wall-perturbing agents and exogenous oxidative stress. Collectively, FonPATs have distinct functions in various basic biological processes, including mycelial growth and morphology, conidiation and conidial morphology, spore germination, and stress response in *Fon*. However, the mechanisms of how the FonPATs regulate the functions in a given basic biological process might be explored through characterizing the palmitoylated substrates for specific FonPATs.

The implication of PATs in fungal pathogenicity has been documented by observations that deletion of *CnPfa4* caused dramatic defects in cryptococcal pathogenicity of C. neoformans (29, 30). Three out of 6 FonPATs, *FonPAT1*, *FonPAT2*, and *FonPAT4*, are required for *Fon* virulence on watermelon, implying that FonPATs and protein palmitoylation are widely involved in *Fon* virulence. As a vascular-colonizing pathogen, effectively penetrating, actively colonizing, and quickly initiating invasive growth and expansion are critical steps for F. oxysporum to successfully cause disease (13, 67). The fact that the growth of *ΔFonpat1* or *ΔFonpat4* in roots was significantly reduced may imply a defect of *ΔFonpat1* or *ΔFonpat4* in penetration and colonization in roots. In contrast, the growth in roots and the penetration ability of *ΔFonpat2* were not affected, while the growth in stems was dramatically decreased. These observations indicate a defect of *ΔFonpat2* in colonizing and spreading within the vascular system of host plants, which can be examined further using GFP- or red fluorescent protein (RFP)-tagged *ΔFonpat2* strains. Therefore, it is possible that *FonPAT1*, *FonPAT2*, and *FonPAT4* may function in different infection stages of *Fon* on watermelon. This was further supported by the downregulation of infection-related genes, including *FonFow2*, *FonSho1*, *FonFvs1*, and *FonMsb*2 (50–52) in *ΔFonpat1*, *ΔFonpat2*, and *ΔFonpat4*.

The functions of PATs depend on their activity to catalyze the palmitoylation of distinct sets of substrates associated with specific pathways. Therefore, characterization of the palmitoylated substrates is a critical issue for understanding the molecular mechanisms of FonPAT1, FonPAT2, and FonPAT4 in the regulation of virulence in *Fon*. The comparative proteomics study identified a total of 211 putative FonPAT2-palmitoylated substrate proteins, and some have homologs that are known to be palmitoylated in other fungal species; e.g., homologs of FOXG_04579 (actin γ) and FOXG_00122 (Ca^2+^-transporting ATPase) were previously reported to be palmitoylated in A. nidulans (33). Importantly, several the FonPAT2-palmitoylated substrates have homologues that have been shown to function in pathogenicity of fungi, e.g., the AP-2 complex subunits (FOXG_00780 and FOXG_04448) and autophagy-related proteins (FOXG_05141 and FOXG_01866) (41, 42, 51–53). This implies the general significance of protein palmitoylation in regulating the virulence of plant-pathogenic fungi, which is consistent with the finding that a single PAT, CnPfa4, is responsible for the palmitoylation of a subset of proteins that are critical in the pathogenicity of C. neoformans (30). However, mycelial samples were used to identify the FonPAT2-palmitoylated substrates related to *Fon* virulence in this study. Indeed, a variety of virulence factors are not expressed in mycelia. Further experiments with germinating conidia or invasive mycelia within infected plant tissues as starting samples will characterize novel FonPAT2 substrates that function as virulence factors during the infection process. On the other hand, palmitoylation of FonAP-2α, FonAP-2β, and FonAP-2μ was specifically catalyzed by FonPAT2 but not by FonPAT1, implying the substrate specificity for FonPAT1 and FonPAT2. This is consistent with the previous observations that the yeast ScSwf1, closely related to FonPAT2, was highly specific for its substrates (68). It was also found that human and yeast PATs show distinct substrate specificity (30, 69, 70). In this regard, FonPAT1 and FonPAT4, which were also required for *Fon* virulence, may have distinct mechanisms through catalyzing different sets of substrates to regulate *Fon* virulence. Notably, a weaker palmitoylated band for FonAP-2μ-GFP was visible in *ΔFonpat2*, indicating that FonAP-2μ may also be palmitoylated by other FonPATs. Furthermore, the palmitoylated FonAP-2μ-GFP band seemed to be stronger in Δ*Fonpat1* than that in the complementation strain Δ*Fonpat1*-C, raising the possibility that FonPAT1 and FonPAT2 may play an opposite role or compete with the substrates. This can be clarified by analyzing the palmitoylation of FonAP-2μ in other mutant strains, including *ΔFonpat4*.

Like the function of the FgAP-2 complex in F. graminearum (41, 42), the FonAP-2 complex is essential for vegetative growth and hyphal morphology, conidiation and conidial morphology, cell wall integrity, and virulence in *Fon*. In particularly, disruption of each of the FonAP-2 complex subunits resulted in defects in hyphal polarized growth and cell wall stress response, which is in agreement with the observations that the AP-2 complex is involved in regulating cell wall integrity in F. graminearum and yeast (40, 42). The facts that FonPATs were involved in oxidative stress response and that the FonAP-2 complex subunits and their palmitoylation are critical to the cell wall integrity may imply a relationship among the FonPAT2-mediated palmitoylation of FonAP-2 complex subunits, cell wall integrity, and oxidative stress response in *Fon*. Notably, the defects observed in *ΔFonpat2* showed similar defects in hyphal polarized growth and cell wall stress response. These similarities, together with the impaired virulence in *ΔFonpat2* and the deletion mutants of the FonAP-2 complex subunits, establish a functional link between FonPAT2 and the FonAP-2 complex in regulating vegetative growth, conidiation, cell wall integrity, and virulence in *Fon*.

The heterotetrameric AP-2 complex is formed by the interactions of the subunits α, β, and μ (71, 72). The interactions of FonAP-2β with FonAP-2α and FonAP-2μ demonstrated the formation of the FonAP-2 complex in *Fon*. The FonAP-2 complex subunits but not their palmitoylation-deficient variants were palmitoylated by FonPAT2 but not by FonPAT1, demonstrating that FonAP-2α, FonAP-2β, and FonAP-2μ are the specific substrates of FonPAT2. Generally, PAT-mediated palmitoylation often regulates dynamic subcellular localization, stability, trafficking, and protein-protein interactions and therefore is essential to maintain protein function (17–21). FonPAT2-catalyzed palmitoylation of FonAP-2α, FonAP-2β, and FonAP-2μ did not affect the subcellular localization of the FonAP-2 complex but is required for the interactions of FonAP-2β with FonAP-2α and FonAP-2μ and the stability of FonAP-2α, FonAP-2β, and FonAP-2μ. This is consistent with the observations that yeast ScSwf1 and apple MdPAT16 stabilized the substrates SNARE Tlg1 and MdCBL1, respectively, through palmitoylation (73, 74). Therefore, it is most likely that the function of FonPAT2 mainly contributes to maintaining the stability and interaction ability of the core subunits FonAP-2α, FonAP-2β, and FonAP-2μ through palmitoylation to enable the formation of the FonAP-2 complex. However, the FonAP-2μ^C24S^-GFP and FonAP-2α^C400S^-GFP proteins were detected, but the interactions of FonAP-2β-FLAG with FonAP-2μ^C24S^-GFP and FonAP-2α^C400S^-GFP were not observed in the co-IP assays using mycelia without the treatment of MG132 or other inhibitors of protein degradation pathways. It is thus worthwhile to investigate the pathway and mechanism responsible for the degradation of the nonpalmitoylated FonAP-2 complex subunits.

**FIG 9:**
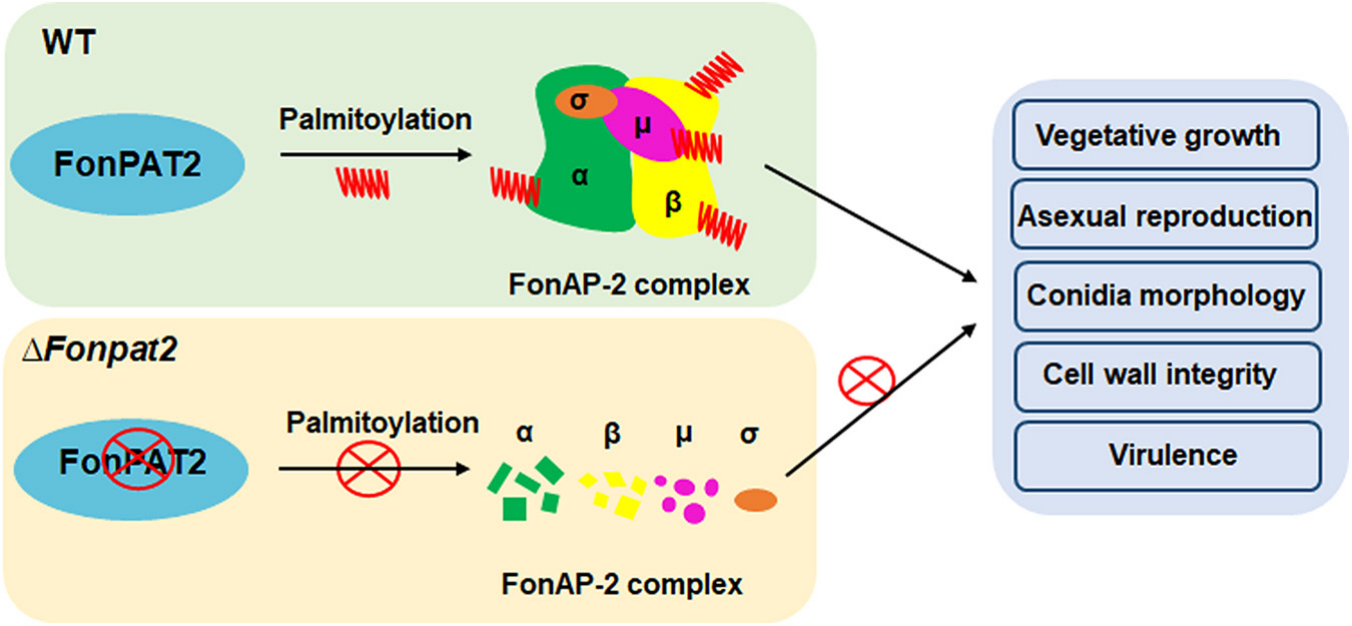
A proposed working model for the functions of the FonPAT2-FonAP-2 complex in *Fon.*

In conclusion, the present study revealed the distinct functions of FonPATs in vegetative growth and mycelial morphology, asexual reproduction, and stress response, as well as the importance of FonPAT1, FonPAT2, and FonPAT4 in virulence in *Fon*. FonPAT2 catalyzes the palmitoylation of a subset of proteins, some of which are involved in fungal pathogenicity. The core subunits of the FonAP-2 complex are the FonPAT2 substrates, and FonPAT2-mediated palmitoylation maintains the stability and interaction ability of the core subunits to ensure the formation of the FonAP-2 complex, which contributes to regulating vegetative growth, asexual reproduction, cell wall integrity, and virulence in *Fon* (Fig. 9). Understanding the molecular and biochemical mechanisms of how FonPAT2 regulates different biological processes, including virulence in *Fon*, will benefit from further detailed and global investigations of the functions of other substrates.

## MATERIALS AND METHODS

### *Fon* strains, plant materials, and growth conditions.

*Fon* race 1 strain ZJ1 was used as a WT strain for fungal transformation, gene-targeted knockout, and complementation experiments (46). *Fon* strains with different genotypes were routinely cultivated on PDA or MM at 26°C under constant fluorescent light (46). *Fon* strains were grown in mung bean liquid broth (20 g/L mung bean, boiled for 20 min, pH 7.0), and spore suspension inoculum (5 × 10^6^ spores/mL) was prepared as previously described (46). Watermelon (Citrullus lanatus) cv. Zaojia was used for the disease assays. Plants were grown in a soil mix (clay to soil, 3:1) in a growth room under fluorescent light (200 μE m^2^ s^−1^) at 22 to 24°C with 70% relative humidity (RH) and a 14-h light/10-h dark cycle.

### Generation of targeted deletion mutants and complementation strains.

The double-joint PCR method was used to prepare the targeted replacement constructs for the genes of interest (44). The upstream and downstream fragments of the target genes were amplified with gene-specific primers (Table S2), and the PCR products were transformed into protoplasts of *Fon* strains (46). Putative positive transformants were selected on PDA plates supplemented with 100 mg/mL hygromycin B and identified by PCR with gene-specific primer pairs (Table S2), followed by further confirmation using Southern blotting. For construction of complementation vectors, fragments containing an ~1.5-kb native promoter region and ORF (without stop codon) of the genes were cotransformed with XhoI-digested vector pYF11 into yeast strain XK-125 using the alkali-cation yeast transformation kit (MP Biomedicals, Solon, OH, USA), yielding the GFP-tag fused vectors. The recombined vectors were transformed into protoplasts of the corresponding deletion mutants, and neomycin-resistant transformants were characterized by PCR and examined for GFP signal. Site-specific point mutations in *FonPAT1*, *FonPAT2*, *FonPAT4*, and FonAP-2 complex subunits *FonAP-2α*, *FonAP-2β*, and *FonAP-2μ* were created using the Mut Express MultiS fast mutagenesis kit (Vazyme Biotech, Nanjing, China) and cloned into pYF11 for construction of complementation strains (46).

### Measurements of basic biological parameters and morphological observation.

Experiments for growth phenotype, conidiation, stress response, and morphological observation were carried out as described previously (44, 46). For growth phenotype, the *Fon* strains were grown on PDA or MM for 7 days, and the colony diameters were recorded. For stress response assays, the *Fon* strains were grown on PDA supplemented with 0.7 mol/L NaCl, 0.7 mol/L CaCl_2_, 0.2 g/L CR, 0.2 g/L CFW, 0.3 g/L SDS, 0.2% H_2_O_2_, or 5% paraquat, and colony diameters were measured at 7 days, followed by calculation of the mycelial growth inhibition rate as previously described (46). A heatmap showing the inhibition rates of the mycelial growth was generated in GraphPad Prism software using the means of the inhibition rates from three independent experiments. For the conidiation assay, the *Fon* strains were cultivated in MBL broth, and the macroconidia were counted at 2 days using a hemocytometer. Macroconidia were stained with 10 μg/mL CFW and observed under a confocal microscope. Macroconidia were allowed to germinate in YEPD (3 g yeast extract, 10 g peptone, and 20 g dextrose in 1 L, pH 7.0) with shaking at 26°C for 12 h and microscopically examined for germination with at least 100 randomly selected macroconidia per field. For the protoplast-releasing assay, fresh mycelia were collected from 2-day-old culture grown in potato dextrose broth (PDB) and treated with an enzymatic solution containing equal amounts of cellulase, lysozyme, and driselase for 2 h at 30°C. The released protoplasts were counted microscopically, and photographs were taken.

### Disease assays and fungal growth estimation.

Disease assays were performed by inoculating 3-week-old watermelon plants with spore suspension of *Fon* strains using a previously reported procedure (44). Plants were inoculated by placing the roots into *Fon* spore suspension or distilled sterilized water as mock controls for 15 min and were then replanted in soil and allowed to grow under the same growth conditions. Disease development and progress were examined and scored using a 4-scale rating standard (46): 0 = no symptoms, 1 = chlorosis, 2 = wilting, and 3 = death. Vascular necrosis in diseased plants was examined under a TM-1000 microscope (Hitachi, Tokyo, Japan). The tissue-based examination and qPCR-based quantification of *in planta* fungal growth in roots and stems were carried out as previously described (44, 75).

### Reverse transcriptase quantitative (RT-qPCR) analysis.

Total RNA was extracted using RNA isolator reagent (Vazyme, Nanjing, China) and reverse transcribed into cDNA using a HiScript QRT SuperMix kit (Vazyme, Nanjing, China) according to the manufacturer’s instructions. Quantitative PCR (qPCR) reactions contained 10 μL 2× AceQ qPCR SYBR green master mix (Vazyme), 0.1 μg cDNA, and 7.5 pmol of each of the gene-specific primers (Table S2) in a final volume of 20 μL and was run in a CFX96 real-time PCR detection system (Bio-Rad, Hercules, CA, USA). *FonActin* was used as an internal control to normalize the data, and the relative expression of the genes tested was calculated using the 2^–ΔΔ^*^CT^* method.

### Fluorescent microscopic observations.

For the subcellular localization assay, fresh mycelia were stained with ER marker ER-Tracker Green or mitochondrial marker MitoTracker Green (Yeasen Biotech, Shanghai, China). The stained mycelia were observed under a LSM780 confocal microscope (Zeiss, Gottingen, Niedersachsen, Germany). Signal was visualized with an excitation wavelength at 488 nm (50% power; pinhole, 90 μm; master gain, 580) for GFP fluorescence or at 561 nm for mCherry fluorescence.

### Complementation assays in yeast *akr1*.

The ORFs (without stop codon) of *FonPAT1*, *FonPAT2*, and *FonPAT4*, and their DHHC-mutated variants *FonPAT1*^C117S^, *FonPAT2*^C99S^, and *FonPAT4*^C475S^ were amplified using gene-specific primers (Table S2) and cloned into pYES2 vector. The recombinant vectors were transformed separately into the yeast *akr1* strain. Empty pYES2 was transformed into WT and *akr1* strains as positive and negative controls, respectively. Complementation assays were performed as previously described (75). Yeast strains were grown on YPRG (10 g/L yeast, 20 g/L peptone, 20 g/L d-galactose, pH 6.7) medium for 2 days at 25°C or 37°C and then transferred to 28°C for 1 day, followed by the examination of their growth performance.

### ABE assays.

ABE assays were performed as previously described, with minor modifications (33, 55). Mycelia of the WT and *ΔFonpat2* strains were ground with liquid nitrogen and resuspended in 2.5 mL lysis buffer (1× phosphate-buffered saline [PBS], 1 mmol/L EDTA, 1% Triton X-100, pH 7.4) containing 250 μL 25× protease inhibitor cocktail (Roche, Mannheim, Germany) and 25 mmol/L freshly prepared *N*-ethylmaleimide solution (Thermo Fisher Scientific, Waltham, MA, USA). The samples were incubated in a rotator at 4°C for 1 h and then centrifuged at 13,000 × *g* at 4°C for 20 min. The supernatant was added with 1 mL 50 mmol/L *N*-ethylmaleimide solution and incubated overnight at 4°C. After the addition of methanol/chloroform/sterilized water (vol/vol/vol = 3:1:4), proteins were collected by centrifugation at 10,000 × *g* at 14°C for 30 min. The pellet was resuspended in 200 μL resuspension buffer (1× PBS, 8 mol/L urea, 2% SDS, pH 7.4) and dissolved in an ultrasonic water bath. One half of the sample was added into 800 μL *N,N*-dimethylformamide (DMF) solution containing 1 mol/L hydroxylamine, 1 mmol/L EDTA, 40 μL 25× protease inhibitor cocktail, and 100 μL 4 mmol/L biotin-HPDP (Thermo Fisher Scientific). The remaining half was added into DMF solution without hydroxylamine and served as a negative control. Proteins were collected by centrifugation at 10,000 × *g* at 14°C for 30 min and resuspended in 100 μL resuspension buffer. The two samples were added with 900 μL PBS containing 0.2% Triton X-100, and 100 μL each of the solutions was taken as loading controls. The remaining sample was incubated with 20 μL streptavidin-agarose beads (Thermo Fisher Scientific) for 1 h and washed four times with 1 mL washing buffer (1× PBS containing 0.5 mol/L NaCl and 0.1% SDS), and proteins were eluted by heating at 95°C in 50 μL 4× SDS sample buffer.

### LC-MS analysis.

After processing with the ABE protocol, proteins were reduced by adding dithiothreitol (DTT) to a final concentration of 10 mmol/L and incubated at 37°C for 1.5 h, followed by alkylation in the dark for 40 min with 50 mmol/L iodoacetamide. After digestion at 37°C overnight with trypsin (trypsin to proteins,1:50, [wt/wt]), the reactions were stopped by adding trifluoroacetic acid, and the peptides were desalted on C_18_ cartridges (Sigma-Aldrich, St. Louis, MO, USA). After concentration by vacuum centrifugation, the peptides were resuspended in 40 μL 0.1% formic acid, and the peptide concentration was estimated by UV spectrometry at 280 nm. LC-MS/MS analysis was performed on a Q Exactive mass spectrometer (Thermo Fisher Scientific, Waltham, MA, USA). MS data were acquired using a data-dependent top 10 method dynamically choosing the most abundant precursor ions from the survey scan (300 to 1,800 *m/z*) for high-energy collisional dissociation (HCD) fragmentation. The raw data files were searched using the MASCOT engine 2.2 (Matrix Science, London, UK) embedded into Proteome Discoverer 1.4 (Thermo Electron, San Jose, CA, USA) against the UniProt database. Thr CSS-Palm 4.0 algorithm (http://csspalm.biocuckoo.org/) was used to predict putative palmitoylation sites in proteins with a medium threshold stringency for performance evaluation.

### Protein extraction and Western blotting.

Approximately 300 mg fresh mycelia were quickly ground with liquid nitrogen and resuspended in 1 mL lysis buffer (50 mmol/L Tris-HCl, 100 mmol/L NaCl, 5 mmol/L EDTA, 1% Triton X-100, 10 μL phenylmethylsulfonyl fluoride [PMSF], pH 7.5) containing 40 μL 25× protease inhibitor cocktail. After homogenization, the suspension was centrifuged at 12,000 × *g* at 4°C for 15 min, and the supernatant was collected. The Western blotting assay was performed as previously described (46). Proteins were separated on 12.5% SDS-PAGE gels and transferred to an Immobilon-P transfer membrane (Millipore, Billerica, MA, USA). Anti-GFP antibody (ab290, Abcam, Cambridge, UK), anti-mCherry antibody (ab125096, Abcam) and anti-GAPDH antibody (Hangzhou HuaAn Biotech, Hangzhou, China) were used at a 1:1,000 to 1:10,000 dilutions for immunoblot analysis. Quantification of Western blotting bands was done using ImageJ software.

### Co-IP assays.

Fragments (native promoters and ORFs) of *FonAP-2α*, *FonAP-2β*, *FonAP-2μ*, and the palmitoylation-defeat variants *FonAP-2α*^C400S^ and *FonAP-2μ*^C24S^ were inserted into vectors pYF11 (GFP tag) or pHZ126 (3×FLAG tag), which were then cotransformed into the WT. The strains bearing FonAP-2α-GFP plus FonAP-2β-FLAG, FonAP-2α^C400S^-GFP plus FonAP-2β-FLAG, FonAP-2μ-GFP plus FonAP-2β-FLAG, FonAP-2μ-GFP^C24S^ plus FonAP-2β-FLAG, or a single construct were generated and confirmed by their antibiotic resistance, PCR amplification of the target constructs, and Western blotting of the proteins with anti-FLAG or anti-GFP antibodies. For co-IP assays, total proteins were extracted and incubated with GFP-Trap beads (ChromoTek, Planegg-Martinsried, Germany) as previously described (46). The eluted proteins were detected with GFP antibody (ab290, Abcam, Cambridge, UK) or FLAG antibody (Sigma-Aldrich, St. Louis, MO, USA).

### BiFC assays.

BiFC assays were performed as previously described (46). In brief, paired constructs FonAP-2α-YFP^C^/FonAP-2β-YFP^N^, FonAP-2α^C400S^-YFP^C^/FonAP-2β-YFP^N^, FonAP-2μ-YFP^C^/FonAP-2β-YFP^N^, FonAP-2μ^C24S^-YFP^C^/FonAP-2β-YFP^N^, FonAP-2μ-YFP^C^, and FonAP-2μ^C24S^-YFP^C^ were cotransformed into the WT to generate strains FonAP-2α-YFP^C^ plus FonAP-2β-YFP^N^, FonAP-2α^C400S^-YFP^C^ plus FonAP-2β-YFP^N^, FonAP-2μ-YFP^C^ plus FonAP-2β-YFP^N^, and FonAP-2μ^C24S^-YFP^C^ plus FonAP-2β-YFP^N^. The strains bearing FonAP-2β-YFP^N^ plus YFP^C^, FonAP-2α-YFP^C^ plus YFP^N^, FonAP-2α^C400S^-YFP^C^ plus YFP^N^, FonAP-2μ-YFP^C^ plus YFP^N^, or FonAP-2μ^C24S^-YFP^C^ plus YFP^N^ were used as negative controls. The transformants were confirmed by PCR. Yellow fluorescent protein (YFP) signal was excited at 514 nm and detected using a 527 emission filter under a LSM880 confocal microscope (Carl Zeiss AG, Jena, Germany).

### Cell-free protein degradation assays.

The ORFs of *FonAP-2α*, *FonAP-2β*, and *FonAP-2μ*, and their palmitoylation-deficient variants were cloned into pGEX4T-3 or pET28a. Prokaryotic expression of recombinant proteins in Escherichia coli strain BL-21 was induced by adding IPTG (isopropyl-β-D-thiogalactopyranoside; Sigma-Aldrich, St. Louis, MO, USA) to a final concentration 0.1 mmol/L, and recombinant proteins were purified using a glutathione S-transferase (GST) fusion protein purification kit (Genscript, Nanjing, China) or Profinity IMAC Ni-charged resin (Bio-Rad, Hercules, CA, USA) according to the manufacturer’s instructions. Cell-free protein degradation assays were carried out following a previously described protocol with modifications (76). Briefly, total proteins were extracted in degradation buffer (5 mmol/L DTT, 10 mmol/L ATP, 4 mmol/L PMSF, 10 mmol/L MgCl_2_, 25 mmol/L Tris-HCl, and 10 mmol/L NaCl) from the WT and *ΔFonpat2* strains and then incubated with GST-FonAP-2α, HIS-FonAP-2β, GST-FonAP-2μ, GST-FonAP-2α^C400S^, HIS-FonAP-2β^C5,260S^, or GST-FonAP-2μ^C24S^ at 22°C. Protein samples were collected, boiled, separated on 12.5% SDS-PAGE gels, and detected by Western blotting using anti-GST and anti-HIS antibodies (1:1,000 dilution; Genescript). GAPDH was detected with anti-GAPDH antibody and used as a control.

### Statistical analysis.

All experiments were independently performed at least three times, and data were subjected to statistical analysis using Student’s *t* test or one-way analysis of variance (ANOVA). A significant difference was defined by the probability values of *P < *0.05.

### Data availability.

The gene ID (FonPAT1, FOXG_11304; FonPAT2, FOXG_06235; FonPAT3, FOXG_07613; FonPAT4, FOXG_02420; FonPAT5, FOXG_03335; FonPAT6, FOXG_03646; FonAP-2α, FOXG_00780; FonAP-2β, FOXG_08330; FonAP-2μ, FOXG_04448; FonAP-2σ, FOXG_08592) of the study can be found at the FungiDB website (https://fungidb.org/fungidb/).
