# Supplementary material for: Palmitoyl Transferase FonPAT2-Catalyzed Palmitoylation of the FonAP-2 Complex Is Essential for Growth, Development, Stress Response, and Virulence in Fusarium oxysporum f. sp. niveum
Source: Microbiol Spectr. 2022 Dec 19;11(1):e03861-22. doi: 10.1128/spectrum.03861-22 (PMC9927311; doi:10.1128/spectrum.03861-22)
Supplement: Supplemental file 1 — Supplemental material. Download spectrum.03861-22-s0001.pdf, PDF file, 2.1 MB [file spectrum.03861-22-s0001.pdf]

Supplemental Materials

Palmitoyl transferase FonPAT2-catalyzed palmitoylation of the FonAP-2 complex is essential for growth, development, stress response and virulence in *Fusarium oxysporum* f. sp. *niveum*

Xiaohui Xiong, Yizhou Gao, Jiajing Wang, Hui Wang, Jiajun Lou, Yan Bi, Yuqing Yan, Dayong Li*, and Fengming Song*

Zhejiang Provincial Key Laboratory of Biology of Crop Pathogens and Insects, MARA Key Laboratory of Molecular Biology of Crop Pathogens and Insects, Institute of Biotechnology, College of Agriculture and Biotechnology, Zhejiang University, Hangzhou 310058, P. R. China

A

Genes	ID in FungiDB	ORF (bp)	Protein (aa)	Cloned ORF (bp)
FonPAT1	FOXG_11304	1290	429	1290
FonPAT2	FOXG_06235	963	320	963
FonPAT3	FOXG_07613	1625	546	1625
FonPAT4	FOXG_02420	2157	718	2157
FonPAT5	FOXG_03335	2037	678	2037
FonPAT6	FOXG_03646	1822	496	1822

B

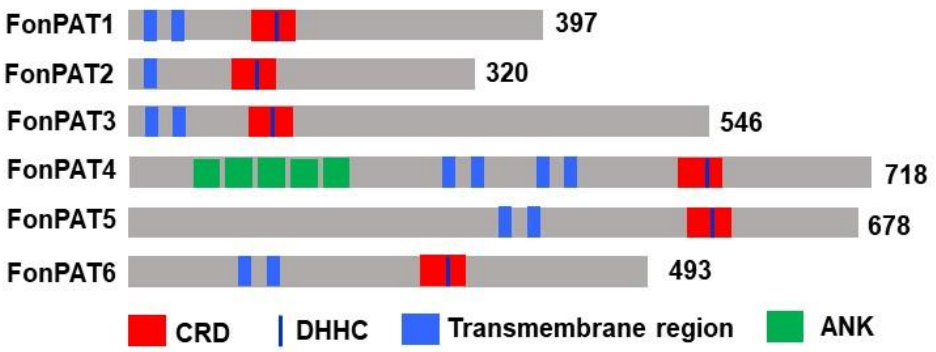

FIG S1 Characterization of *FonPATs* in *Fusarium oxysporum* f. sp. *niveum*. (A) Information on the *FonPAT* genes and proteins in *F. oxysporum* f. sp. *niveum*. (B) Organization of conserved domains in FonPATs. Protein domains were analyzed with SMART protein database (http://smart.embl-heidelberg.de/). CRD, cysteine-rich domain; DHHC, the conserved DHHC motif; ANK, ankyrin-repeat domain.

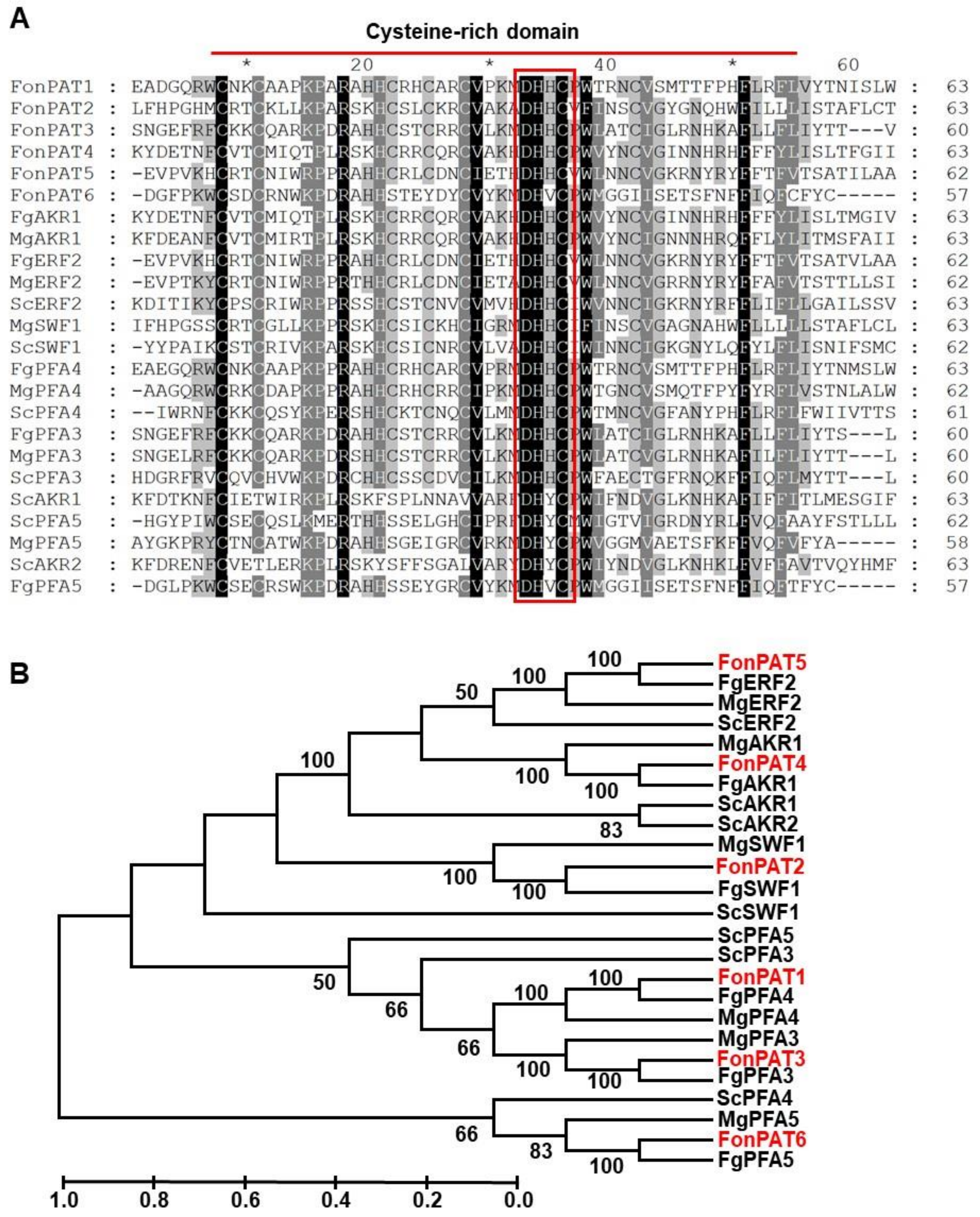

FIG S2 Sequence alignment of the cysteine-rich domains and phylogenetic tree of FonPATs. (A) Amino acid alignment of the cysteine-rich domains in FonPATs and PATs from other fungi. CRD domain is indicated and DHHC motifs are boxed with red lines. (B) Phylogenetic analysis of *Fusarium oxysporum* f. sp. *niveum* FonPATs with PATs from *Saccharomyces cerevisiae* (Sc), *Magnaporthe oryzae* (Mg), and *Fusarium graminearum* (Fg). The phylogenetic tree was constructed by Mega 5.0 using the neighbour-joining method. The bootstrap values from 1000 replications are indicated on the branches. The NCBI accession numbers of the yeast PATs are: ScERF2, YLR246W; ScAKR1, YDR264C; ScAKR2, YOR034C; ScSWF1, YDR126W; ScPFA5,

YDR459C; ScPFA3, YNL326C; and ScPFA4, YOL003C. The locus numbers for FonPATs include FonPAT1, FOXG_11304; FonPAT2, FOXG_06235; FonPAT3, FOXG_07613; FonPAT4, FOXG_02420; FonPAT5, FOXG_03335; and FonPAT6, FOXG_03646. The locus numbers of putative PATs in *M. oryzae* are MgERF2, MGG_03664; MgAKR1, MGG_07382; MgSWF1, MGG_00810; MgPFA3, MGG_02437; MgPFA4, MGG_00747; and MgPFA5, MGG_02933. The locus numbers of putative PATs in *F. graminearum* include FgERF2, FGSG_08531; FgAKR1, FGSG_06542; FgSWF1, FGSG_09537; FgPFA3, FGSG_05896; FgPFA4, FGSG_01411; and FgPFA5, FGSG_16945). The locus numbers for fungal PATs can be found at the FungiDB database (https://fungidb.org/fungidb/app).

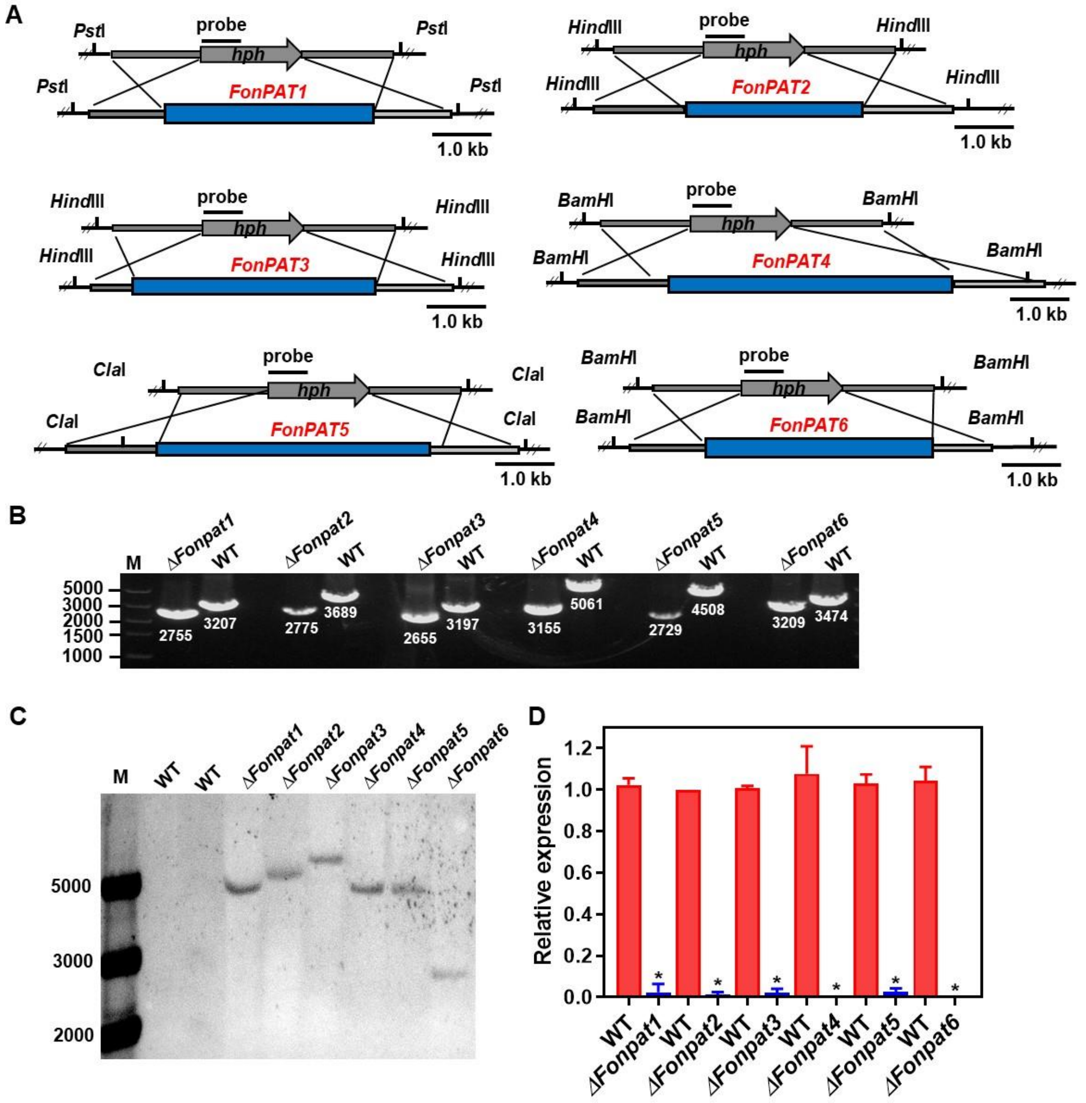

FIG S3 Generation and characterization of the Δ*Fonpat* deletion mutants. (A) Schematic diagrams for the strategies to generate the targeted deletion mutants for each of the *FonPAT* genes. *HPH*, hygromycin B resistance gene cassette. The *HPH* fragment used as a hybridization probe is indicated. (B) PCR identification of the Δ*Fonpat* deletion mutants. The sizes of DNA markers and the amplified fragments from the mutant and WT strains are indicated in bp. (C) Southern blotting validation of the Δ*Fonpat* deletion mutants with the DIG-labeled *HPH* fragment. The sizes of DNA markers are indicated in bp. (D) qRT-PCR analysis of the transcript levels in the Δ*Fonpat* deletion mutants with *FonActin* as an internal control. Experiments were independently performed for three times with similar results. Data presented are the means ±SD from three independent experiments and asterisks above the columns indicate the significant difference at p<0.05 level.

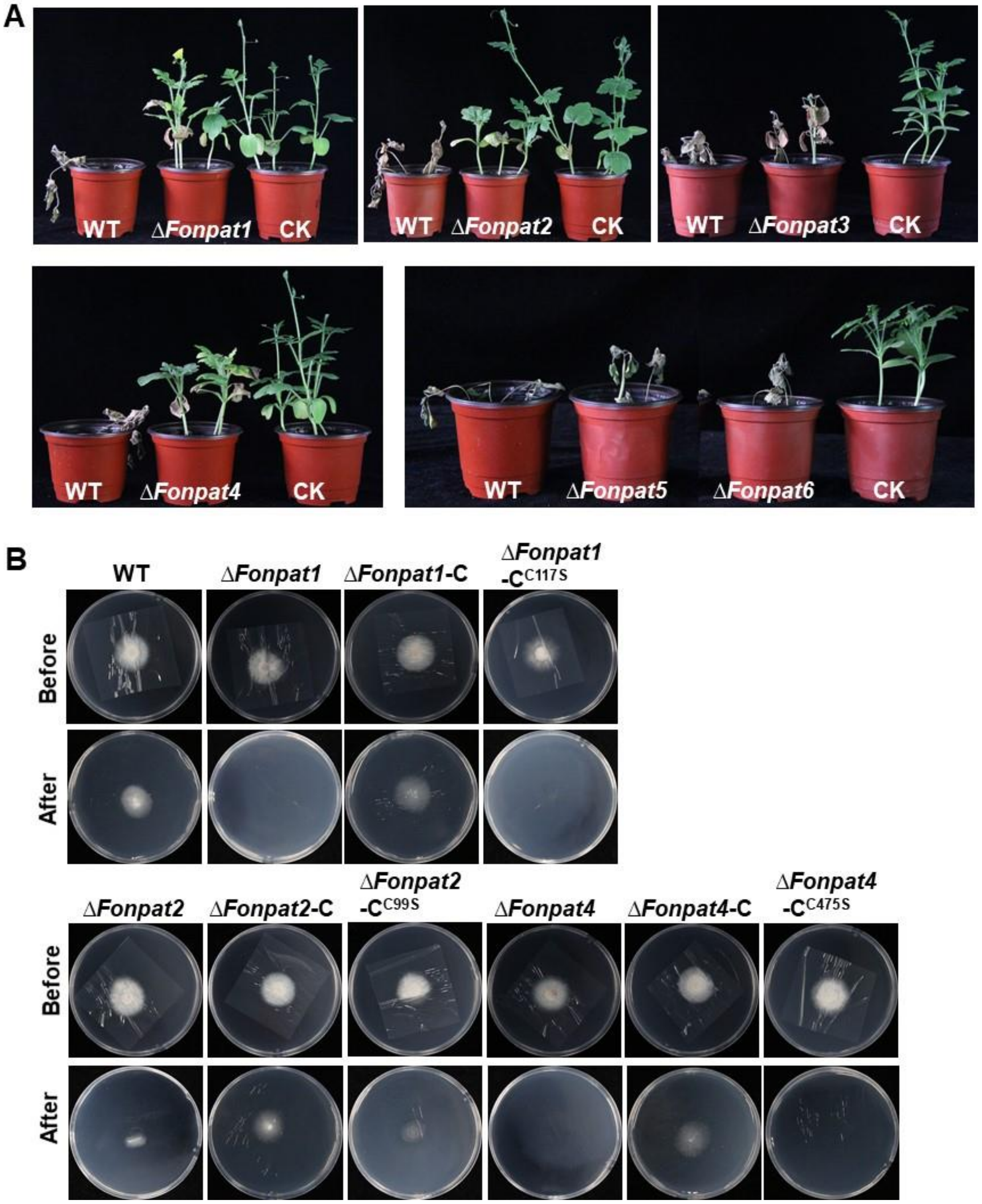

FIG S4 Pathogenicity of the Δ*Fonpat* deletion mutants on watermelon and the significance of the DHHC motifs in FonPAT1 and FonPAT4 for the penetration ability against cellophane membrane. (A) Pathogenicity assays of the Δ*Fonpat* deletion mutants on watermelon. Watermelon plants were inoculated by dipping the roots in spore suspension of the indicated strains and disease phenotype was photographed at 21 days post inoculation. CK, plants inoculated with a preparation of mung bean broth without *Fon* spores. (B) Penetration assays on cellophane membrane. The indicated strains (WT, Δ*Fonpat1*, Δ*Fonpat2*, Δ*Fonpat4*, Δ*Fonpat1-C*, Δ*Fonpat2-C*, Δ*Fonpat4-C*, and DHHC-mutated versions of FonPAT1, FonPAT2, FonPAT4) were grown on cellophane membrane on MM plates for 3 d, and the cellophane membrane along with the fungal colonies were then removed, followed by a 2-d incubation to allow the

growth of mycelia. Experiments were independently performed for three times with similar results.

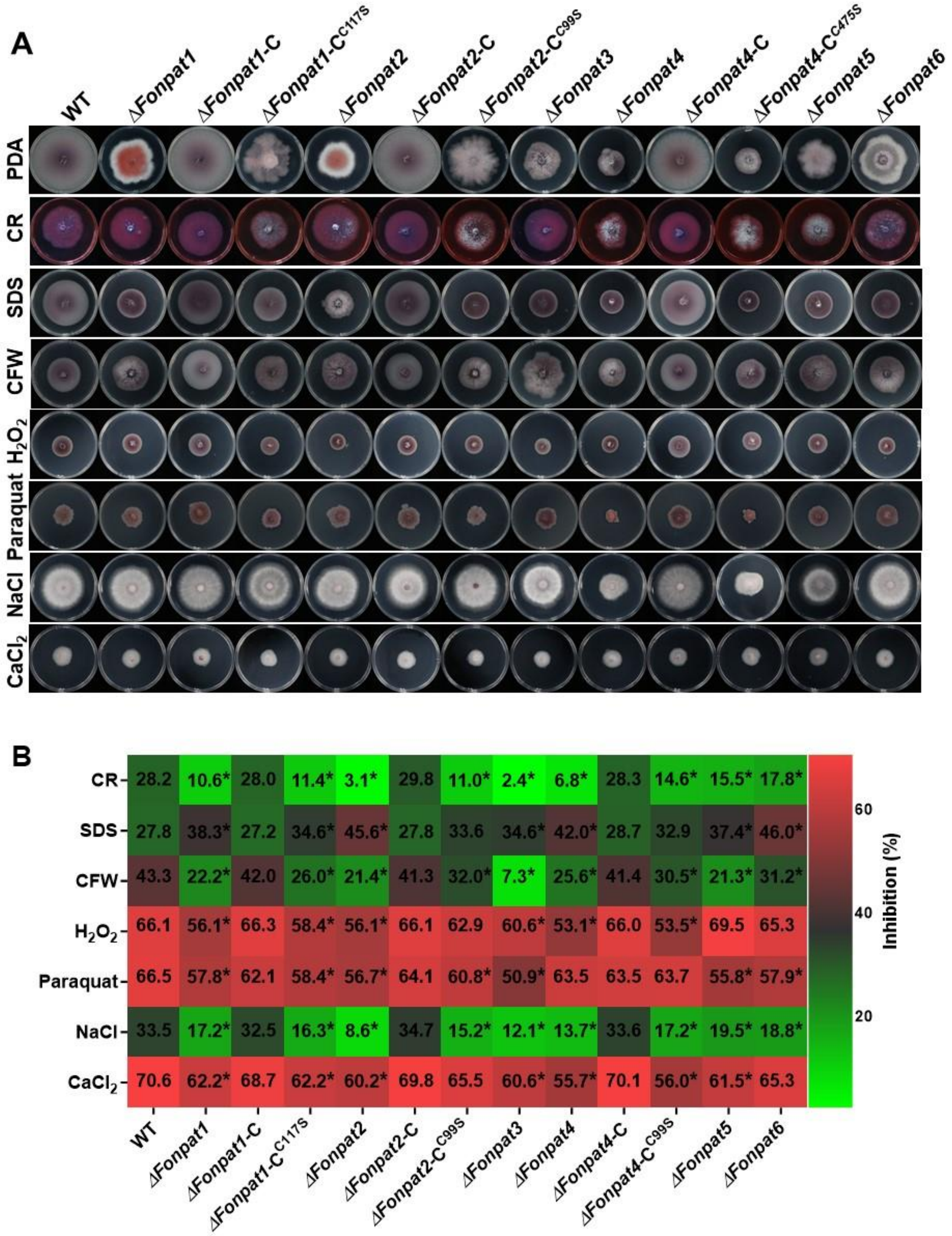

FIG S5 Functions of *FonPATs* in stress response of *Fon*. (A) Growth phenotype and (B) inhibition rates of the mycelial growth of the WT, deletion mutants Δ*Fonpat1*, Δ*Fonpat2*, Δ*Fonpat3*, Δ*Fonpat4*, Δ*Fonpat5*, Δ*Fonpat6*, and complementation strains expressing *FonPAT1*-C, *FonPAT2*-C, *FonPAT4*-C, or DHHC-mutated variants *FonPAT1*-C^{C117S}, *FonPAT2*-C^{C99S}, or *FonPAT4*-C^{C475S} grown on PDA plates supplemented with 0.2 g/L CR, 0.2 g/L CFW, 0.3 g/L SDS, 0.2% H_2O_2, 0.7 mol/L NaCl, 0.7 mol/L $CaCl_2$ and 5% paraquat for 7 d at 26°C. Experiments were independently performed for three times with similar results. Data presented in (B) are the means from three independent experiments and asterisks indicate the significant difference at $p < 0.05$ level.

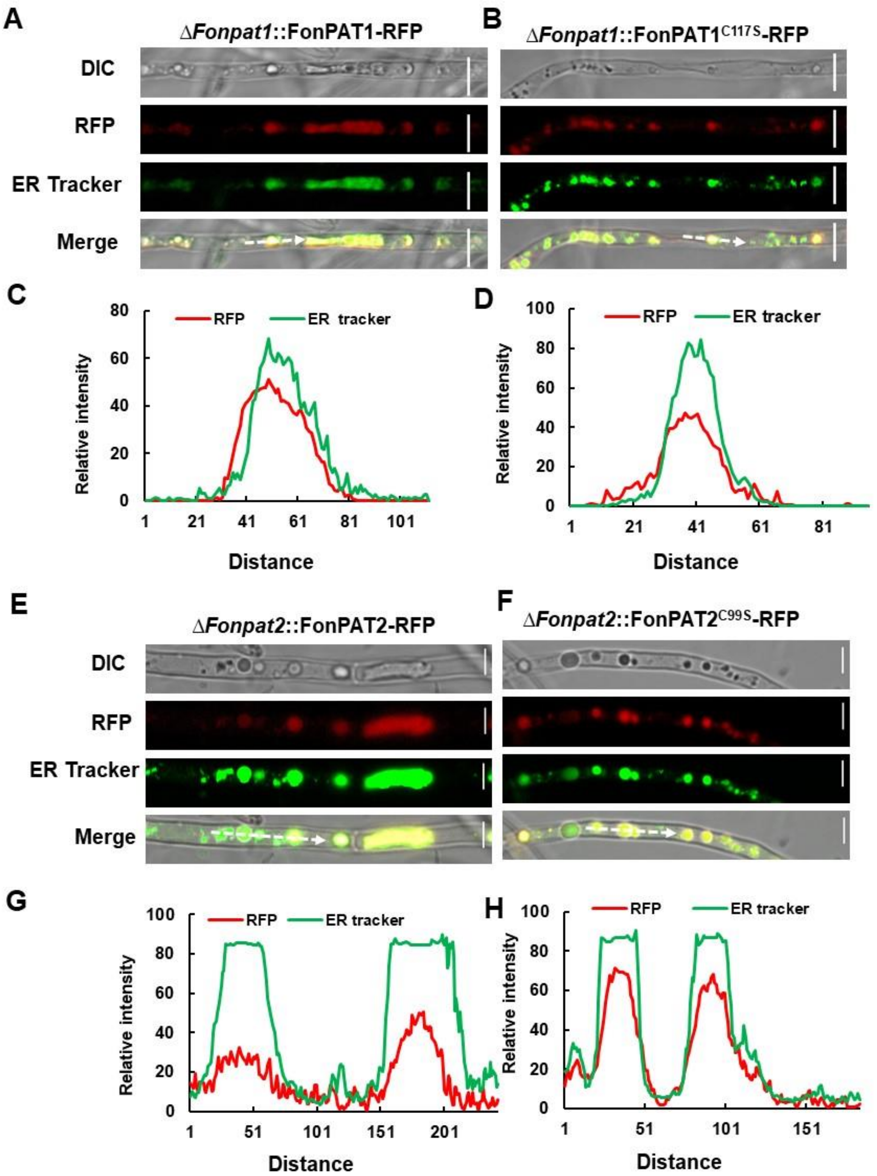

FIG S6 The DHHC motifs are dispensable for the endoplasmic reticulum localization of FonPAT1 and FonPAT2 in *Fon.* Entire ORFs of FonPAT1, FonPAT1^{C117S}, FonPAT2, and FonPAT2^{C99S} were fused with mCherry fragment with their native promoters. All strains were grown in PDB medium for 24 h. (A) and (B) Fluorescent signal from FonPAT1-mCherry or FonPAT1^{C117S}-mCherry and ER-tracker. White line indicated the areas used for linescan graph analysis. Bar=5 µm. (C) and (D) Linescan graphs showing the colocalization of fluorescent signals from FonPAT1-mCherry and FonPAT1^{C117S}-mCherry or GFP ER-tracker. (E) and (F) Fluorescent signal from FonPAT2-mCherry and FonPAT2^{C99S}-mCherry and ER-tracker. White line indicated the areas used for linescan graph analysis. Bar=5 µm. (G) and (H) Linescan graph showing the colocalization of FonPAT2-mCherry and FonPAT2^{C99S}-mCherry and GFP ER-tracker. Photographs were taken using differential interference contrast (DIC) and epifluorescence microscopy. Experiments were independently performed for three times with similar results.

A

Name	Loci	Position	Peptide	Score	Cutoff	LC-MS/MS
FonAP-2α	FOXG_00780	400	LDLLYSMCDATNAQV	5.583	3.717	√
FonAP-2β	FOXG_08330	5	***MLNRCFLFLVNY	15.264	10.722	---
		260	NDIRVFFCKYNDPIY	3.920	3.717	---
FonAP-2μ	FOXG_04448	24	FRAFRNDCRPRLADV	4.692	3.717	√
FonAP-2σ	FOXG_08592	---	---	---	---	---

B

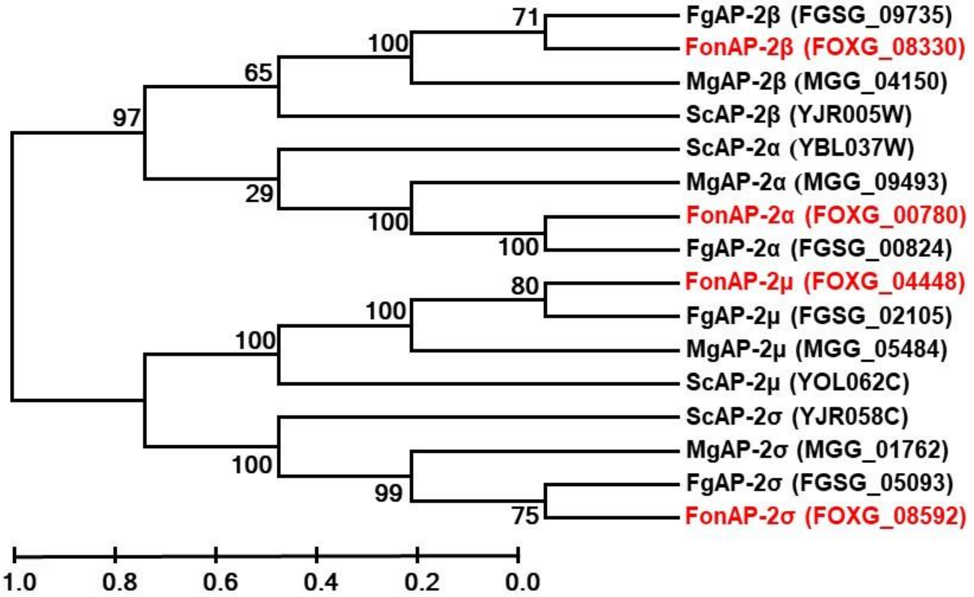

FIG S7 Characterization of the FonAP-2 complex subunits in *Fusarium oxysporum* f. sp. *niveum*. (A) Information on the FonAP-2 complex subunits and their putative palmitoylation sites predicted by CSS-Palm 4.0. Putative palmitoylated cysteines are indicated in red. (B) Phylogenetic relationship of the FonAP-2 complex subunits with orthologous from other species. AP-2 complex subunits used in phylogenetic tree construction included *Magnaporthe oryzae* (Mg), *Saccharomyces cerevisiae* (Sc) and *Fusarium graminearum* (Fg). The phylogenetic tree was constructed with Mega 5.0 using the neighbour-joining method. The bootstrap values from 1000 replications are indicated on the branches. The NCBI accession numbers for yeast AP-2 complex subunits are shown and the locus numbers for the AP-2 complex subunits in *F. oxysporum* f.sp. *niveum*, *M. oryzae*, and *F. graminearum* can be found at the FungiDB database (https://fungidb.org/fungidb/app).

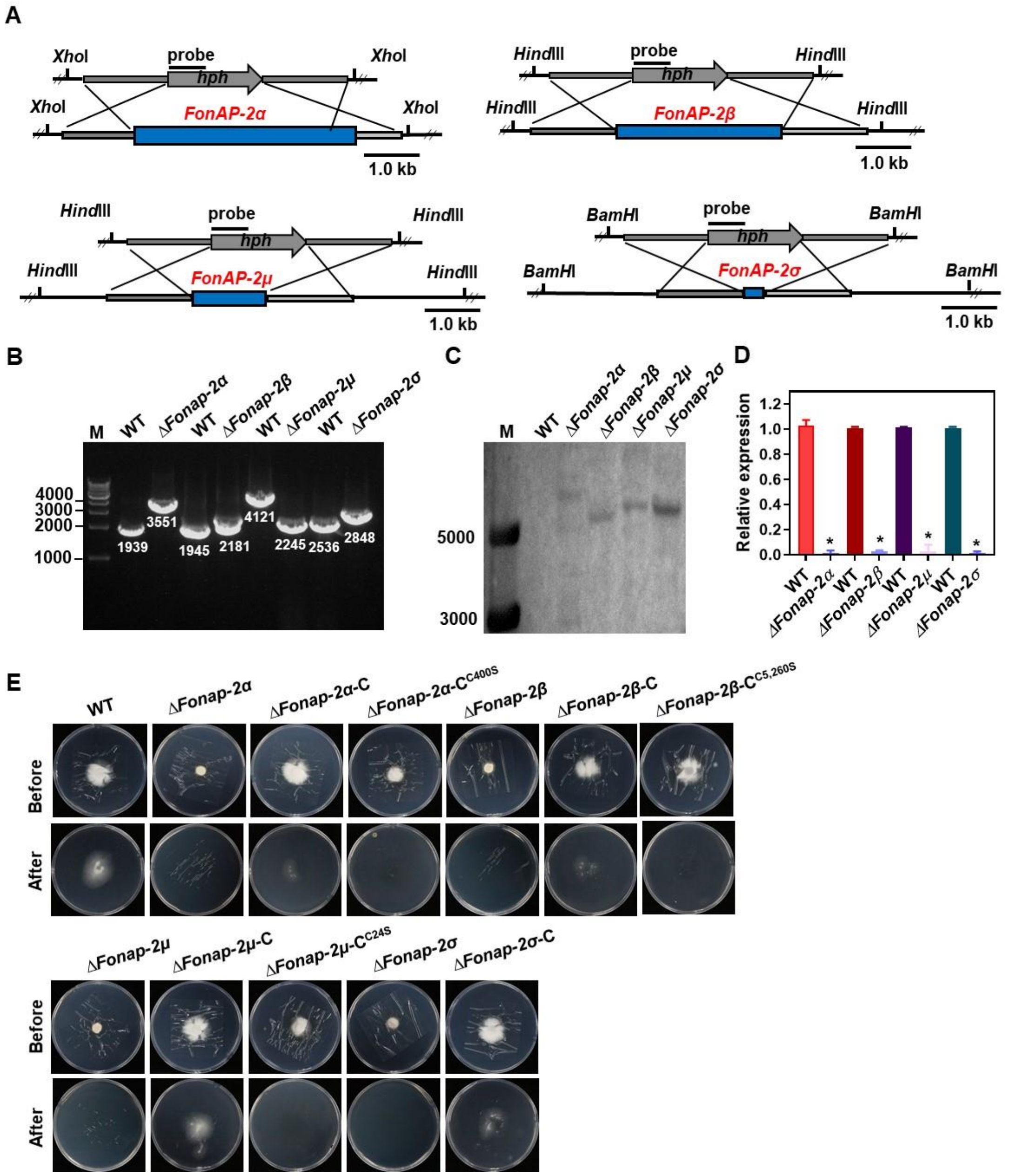

FIG S8 Generation and characterization of the deletion mutants for the FonAP-2 complex subunits and the importance of palmitoylation of FonAP-2α, FonAP-2β, and FonAP-2μ for the penetration ability. (A) Schematic diagrams for the strategies to generate targeted deletion mutants for each of FonAP-2 complex subunits. *HPH*, hygromycin B resistance gene cassette. The *HPH* fragment used as a hybridization probe is indicated. (B) PCR identification of the targeted deletion mutants for the FonAP-2 complex subunits. The sizes of DNA markers and the amplified fragments from the mutant and WT strains are indicated in bp. (C) Southern blotting of the targeted deletion mutants for the FonAP-2 complex subunits. Genomic DNA was digested with corresponding enzymes, separated by electrophoresis, and transferred to NC membranes, followed by hybridization with a DIG-labeled 776 bp *HPH* probe. The sizes of DNA markers are indicated in bp. (D) qRT-PCR analysis of the transcript levels

of the subunit genes of the FonAP-2 complex in the Δ*Fonap-2* deletion mutants. *FonActin* was used as an internal control. (E) Palmitoylation of FonAP-2α, FonAP-2β, and FonAP-2μ is important for the penetration ability of *Fon* against cellophane membrane. Mycelial plugs of the indicated strains were grown on cellophane membrane on MM plates for 3 d at 26℃, and the cellophane membrane along with the fungal colonies were then removed, followed by a 2-d incubation to allow the growth of mycelia. Experiments were independently performed for three times with similar results.

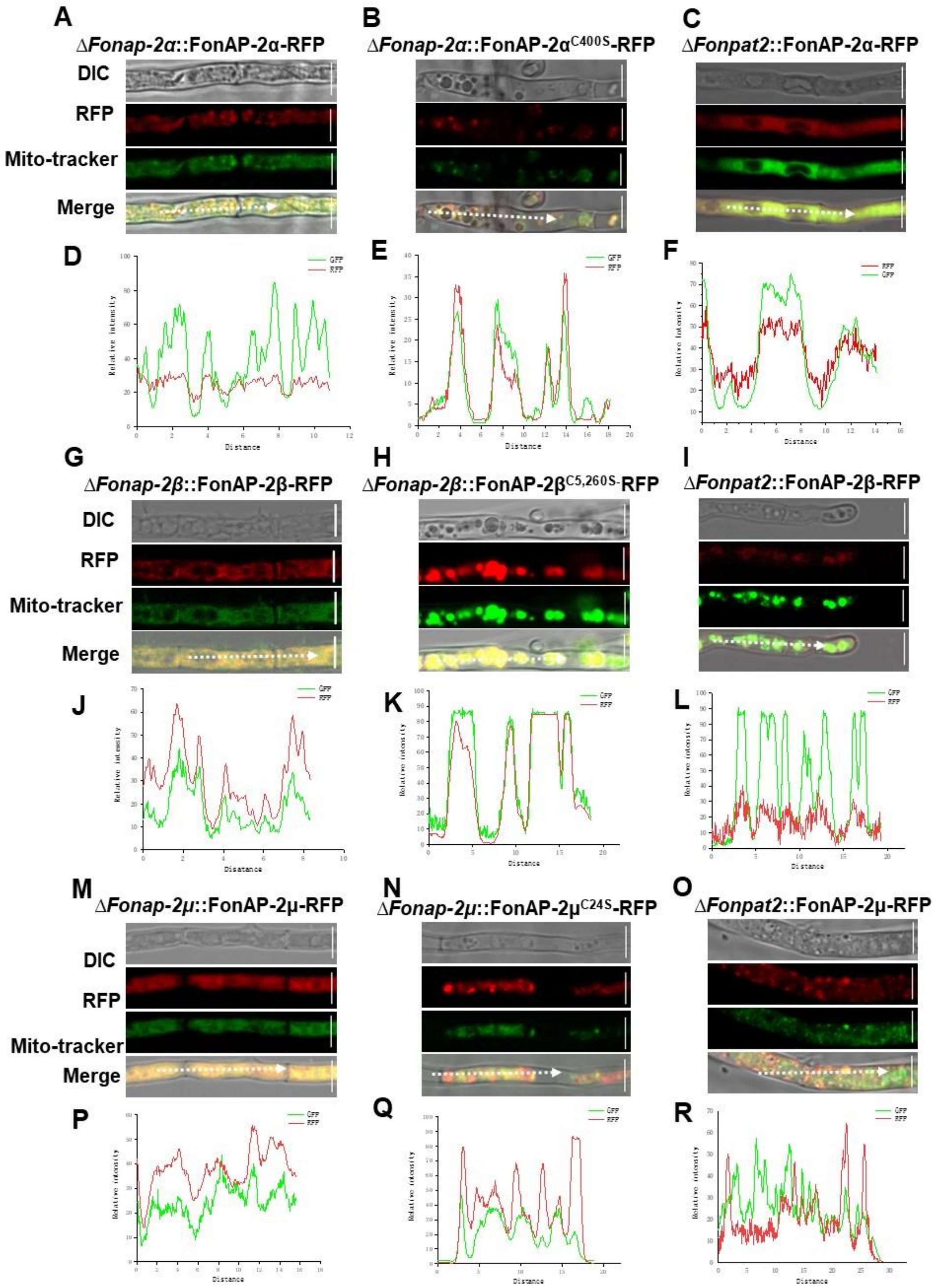

FIG S9 Palmitoylation is not essential for the subcellular localization of FonAP-2α, FonAP-2β, and FonAP-2μ. (A), (B), and (C) Subcellular localization of RFP-tagged FonAP-2α and palmitoylation-defeat FonAP-2α^{C400S} in Δ*Fonap-2α* and Δ*Fonpat2* mutants. Entire ORFs of *FonAP-2α* and *FonAP-2α*^{C400S} were fused with mCherry fragment with its native promoter. (D), (E), and (F) Fluorescence signal from FonAP-2α-RFP or FonAP-2α^{C400S}-RFP and mito-tracker. White arrows indicated the areas used for linescan graph analysis. (G), (H), and (I) Subcellular localization of RFP-tagged FonAP-2β and palmitoylation-defeat FonAP-2β^{C5,260S} in Δ*Fonap-2β* or Δ*Fonpat2*

mutants. Entire ORFs of *FonAP-2β* and *FonAP-2β*^{C5,260S} were fused with mCherry fragment with its native promoter. (J), (K), and (L) Fluorescence signal from FonAP-2β-RFP or FonAP-2β^{C5,260S}-RFP and mito-tracker. White arrows indicated the areas used for linescan graph analysis. (M), (N), and (O) Subcellular localization of RFP-tagged FonAP-2μ and palmitoylation-defeat FonAP-2μ^{C24S} in Δ*Fonap-2μ* or Δ*Fonpat2* mutants. Entire ORFs of *FonAP-2μ* and *FonAP-2μ*^{C24S} were fused with mCherry fragment with its native promoters. (P), (Q), and (R) Fluorescence signal from FonAP-2μ-RFP or FonAP-2μ^{C24S}-RFP and mito-tracker. White arrows indicated the areas used for linescan graph analysis. Bar=5 μm. Experiments were independently performed for three times with similar results.

TABLE S1 FonPAT2-palmitoylated substrates identified by ABE-LC/MS/MS.

	Gene ID	Description	Position	Peptide	Score	Cutoff	Replicate 1	Replicate 2
1	FOXG_01628	vacuolar protein sorting-associated protein 1	511	DLVAMESCYVNTGHP	3.831	3.717	√	√
2	FOXG_00122	Ca^{2+} transporting ATPase	343	SVETLGSCSVICSDK	3.355	2.412	√	√
3	FOXG_00780	AP-2 adaptor complex subunit alpha	400	LDLLYSMCDATNAQV	5.583	3.717	√	√
4	FOXG_00882	transcription elongation factor S-II	301	TFCECMACGHRWKFS	4.365	3.717	√	√
5	FOXG_01952	DNA topoisomerase I	568	NEADTVGCCSLKYEH	2.757	2.412	√	√
			569	EADTVGCCSLKYEHI	4.963	3.717		
6	FOXG_01983	translation initiation factor 2 subunit gamma	136	YKCDNQACPRPGCYR	3.576	2.412	√	√
7	FOXG_02027	RNA binding protein (Arp)	23	VIHVATTCDEHGVYV	4.61	3.717		
			441	FRAGDWKCGNEVCGY	11.114	10.722	√	√
			456	HNFAKNVCCLRCGAS	7.506	2.412		
8	FOXG_02130	related to ribosomal protein L19, mitochondrial	12	SVGRPVGCLKSALRR	16.844	10.722	√	√
9	FOXG_02741	polyketide synthase, putative	1076	NEGWSEHCRGCIAAV	12.777	10.722	√	√
10	FOXG_04178	heat shock 70kDa protein 4	479	GKDDFMICKLKARVN	5.226	3.717	√	√
11	FOXG_04448	AP-2 adaptor complex subunit mu	24	FRAFRNDCRPRLADV	4.692	3.717	√	√
12	FOXG_05141	ubiquitin-like modifier-activating enzyme	623	IRGQSYDCCSACSPK	9.181	2.412	√	√
13	FOXG_05147	NADH-ubiquinone oxidoreductase, mitochondrial	17	RSAIRPVCRAARPQS	23.326	3.717	√	√
14	FOXG_03232	enoyl-[acyl-carrier protein] reductase I	205	NMGTRFMCTVEAPVH	3.758	3.717	√	√
15	FOXG_11547	RING finger domain protein	46	PGSWVDPCGCTLEAH	11.16	10.722	√	√
16	FOXG_05551	ATP dependent RNA helicase, putative	1116	LAIRVKICKIGGIEE	4.892	3.717	√	√
17	FOXG_06208	branched-chain amino acid aminotransferase	23	MSSLRPLCHAQRFSI	11.488	3.717	√	√
			373	WKGKLVDCGLSELEE	11.047	10.722		
18	FOXG_07574	poly [ADP-ribose] polymerase 2	638	SSKSAGYCCSYNTGG	6.814	2.412	√	√
			639	SKSAGYCCSYNTGGE	5.808	3.717		

19	FOXG_08014	protein transporter sec-31	1259	QRDKTEECGNWMVGV	11.661	10.722	√	√
20	FOXG_08526	lon protease like, mitochondrial	44	ACRNHHICGRRLPAI	11.95	10.722	√	√
			1098	KANTCKICEWKAQQK	4.608	3.717		
21	FOXG_11451	Elongation factor 3	279	AVIVDNMCKLVDDPN	6.161	3.717	√	√
22	FOXG_12779	cytochrome c oxidase assembly protein subunit 11	21	AASSQWSCFFCQNTR	18.964	3.717	√	√
			24	SQWSCFFCQNTRPKL	9.511	3.717		
23	FOXG_13835	small GTPase-binding protein	16	VIVGDGACGKTCLLI	23.374	3.717	√	√
24	FOXG_13911	Ras-like protein Rab-11B	211	DAAKGGKCC******	6.616	2.412	√	√
			212	AAKGGKCC*******	15.939	10.722		
25	FOXG_14636	phosphoenolpyruvate synthase	315	LIEEHYSCPMDMEWA	12.351	10.722	√	√
26	FOXG_13733	mannose-6-phosphate isomerase	10	VPLLRLQCGVNSYAW	35.283	3.717	√	√
27	FOXG_12267	MFS transporter, SP family, sugar:H+ symporter	326	GAAWMMMCFFIYAFV	7.269	3.717	√	√
28	FOXG_11323	homoisocitrate dehydrogenase	283	GFAIGEPCHGSAPDI	13.886	10.722	√	√
29	FOXG_11140	ubiquitin conjugation factor E4 B	199	LMNYFLPCWKRAVKA	4.524	3.717	√	√
30	FOXG_10331	50S ribosomal protein L15	13	SPFSTPLCCRTTANA	13.255	2.412	√	√
31	FOXG_09782	siderochrome-iron transporter, putative	451	SIGYLVMCEVFISIA	3.898	3.717	√	√
32	FOXG_09392	vacuolar protein sorting-associated protein 21	235	GAKDSCAC*******	11.013	10.722	√	√
33	FOXG_09462	coatomer subunit delta	9	VVLAASICTRGGKAV	38.323	3.717	√	√
34	FOXG_09061	50S ribosomal protein L4	8	MAGKGIGCLAEAMGA	14.493	10.722	√	√
35	FOXG_08681	glycerol-3-phosphate dehydrogenase [NAD+]	22	KSSRLLSCTHIRTYS	15.689	10.722	√	√
			160	HQFIANVCKQVNGHI	6.217	3.717		
36	FOXG_08700	trehalose 6-phosphate synthase	6	**MTVFVCSLFLPKT	39.704	3.717	√	√
37	FOXG_00015	ATP phosphoribosyltransferase	122	MELGFGSCKLQVQVP	5.479	3.717	√	
38	FOXG_00054	thioesterase family protein	17	GRLAAQACKRFARPT	23.358	3.717	√	
39	FOXG_00577	phospho-2-dehydro-3-deoxyheptonate aldolase	177	RQRVMVDCSHGNSSK	4.239	3.717	√	
40	FOXG_00180	replication factor C subunit 2	178	ITRFCLICNYVTRII	3.787	3.717	√	

41	FOXG_00240	aminomethyltransferase	3	*****MYCTCHVPVL	19.578	10.722	
			5	***MYCTCHVPVLSA	41.106	3.717	√
42	FOXG_00378	heat shock protein 78, mitochondrial	233	LARGELQCCGATTLN	4.929	2.412	√
43	FOXG_00450	anthranilate phosphoribosyltransferase	269	EELDEVSCAGNTLCW	10.911	10.722	√
44	FOXG_00505	ankyrin repeat protein	439	VEDVIEDCCGCCPEA	8.452	2.412	
			440	EDVIEDCCGCCPEAV	4.357	3.717	√
			442	VIEDCCGCCPEAVTD	6.354	2.412	
45	FOXG_00549	hypothetical protein	308	LGSVNVVCTDKTEHG	4.207	3.717	√
			785	DMFNALSCRKGFLAM	5.586	3.717	
46	FOXG_00582	AGC/AKT protein kinase	685	GVLVFEMCCGWSPFY	10.007	2.412	√
			686	VLVFEMCCGWSPFYA	5.406	3.717	
47	FOXG_00681	ubiquinone biosynthesis monooxygenase Coq6	15	LGKPGFVCRSCVRQY	22.263	3.717	√
			18	PGFVCRSCVRQYQYQ	30.027	3.717	
48	FOXG_00729	hypothetical protein	3	*****MDCNANIVIP	14.655	10.722	√
49	FOXG_00003	AMP deaminase, putative	783	KLNSVDMCELAKNSV	6.22	3.717	√
50	FOXG_00950	E3 ubiquitin-protein ligase HUWE1	3540	IESLMVVCKNTTTND	5.63	3.717	√
51	FOXG_00962	DNA-directed RNA polymerase III subunit RPC3	11	TKHAAELCALLVNDL	11.583	10.722	√
52	FOXG_00973	L-iditol 2-dehydrogenase	210	IGLITLQCCAAAGAS	7.148	2.412	√
53	FOXG_00987	chromatin assembly factor 1 subunit B	508	CVVSNLHCATFTDLA	4.169	3.717	√
54	FOXG_01192	Sterol uptake control protein 2	14	HNKSRHGCRNCKKRH	24.015	3.717	
			17	SRHGCRNCKKRHVKC	24.738	3.717	√
			31	CDEGGPPCTNCLARN	13.162	10.722	
55	FOXG_01512	NAD+ synthase (glutamine-hydrolysing)	10	DLITLATCSLNQWVL	37.102	3.717	
			110	ILDGKLLCLRPKIYL	3.717	3.717	√
			373	ATIVFSMCRLVVEAI	8.039	3.717	
56	FOXG_01585	DUF410 domain protein	68	GGSGGDLCAMLVDVY	12.129	10.722	√

57	FOXG_01681	ARF GTPase activator (Csx2), putative	193	GGGWRNSCSFVEYDP	13.618	10.722	√
58	FOXG_01701	chromodomain-helicase-DNA-binding protein 1	462	LRGLNFLCLNWTKGN	4.112	3.717	√
			515	LSVIPAWCDTFNHWS	3.764	3.717	
59	FOXG_01757	splicing factor 3B subunit 1, putative	586	QIPILMGCAVLPHLK	3.932	3.717	√
60	FOXG_01784	glutamate decarboxylase	3	*****MICGLGSNSV	16.53	10.722	
			20	VPVDEFGCMKSGALR	17.747	3.717	√
			215	PPPCLQVCFYYSPGR	3.229	2.412	
61	FOXG_01808	hypothetical protein	18	VRTLTHLCACPDKQV	25.895	3.717	
			20	TLTHLCACPDKQVVL	17.115	3.717	√
			189	KQGVVRMCAFLLQTL	7.585	3.717	
62	FOXG_01857	glycyl-tRNA synthetase	177	DKFADWMCKDPKNGE	3.963	3.717	√
			464	GWIECVGCADRSAYD	4.844	3.717	
63	FOXG_01866	sorting nexin-41	272	SSLAADLCELGSVYN	4.085	3.717	√
64	FOXG_01869	paf1 complex subunit	115	RDKVSMICCLCWMYL	4.135	2.412	
			116	DKVSMICCLCWMYLW	9.965	3.717	√
			118	VSMICCLCWMYLWKS	6.004	3.717	
			263	DPRIGIGCCFWQLGF	7.212	2.412	
			264	PRIGIGCCFWQLGFK	4.784	3.717	√
65	FOXG_01888	adenosine kinase	21	LPSLLPSCCRTCTRN	11.132	2.412	
			22	PSLLPSCCRTCTRNF	12.588	3.717	√
			25	LPSCCRTCTRNFSSF	4.094	3.717	
66	FOXG_01944	histidyl-tRNA synthetase	387	LKERMSVCAKLWESG	3.953	3.717	√
67	FOXG_01948	lysine-specific histone demethylase 1	248	AQTLRLLCIYSCISG	3.985	2.412	√
68	FOXG_19023	chitin synthase	784	LGRTGIMCCFSRR**	14.346	2.412	√
			785	GRTGIMCCFSRR***	6.248	3.717	
69	FOXG_01976	pre-mRNA-splicing ATP-dependent RNA helicase	350	RVLVLAQCCYVIMDE	4.505	2.412	√

70	FOXG_02086	phospholipid-translocating ATPase	623	DKTGTLTCNQMEFKQ	3.8	3.717	√
			974	FLDLAIMCKAVICCR	4.31	3.717	
			979	IMCKAVICCRVSPLQ	14.274	2.412	
			980	MCKAVICCRVSPLQK	8.938	3.717	√
			1269	TVCLAIMCLLRDFVW	5.668	3.717	
71	FOXG_02126	enoyl-CoA hydratase	6	**MNTFRCMRPVAAR	37.841	3.717	√
72	FOXG_02149	glutathione synthetase	509	GVAAGFGCMDSCALV	13.445	10.722	√
			513	GFGCMDSCALV****	11.449	10.722	
73	FOXG_02171	pre-mRNA-splicing factor RSE1	429	AMNPLLDCKVANLTG	4.168	3.717	√
			1088	PHRVSLMCHFYTQDI	3.842	3.717	
74	FOXG_02193	glutathione S-transferase	7	*MGGRIDCYLDIVSF	37.062	3.717	√
75	FOXG_02232	mitochondrial ribosomal protein L43	23	LGAFILQCKKLDFYY	4.735	3.717	√
76	FOXG_02267	3-isopropylmalate dehydrogenase	4	****MGGCSINANGT	12.584	10.722	√
77	FOXG_02274	hypothetical protein related to RAY38 protein	335	ESAPKIPCLHPLTA*	11.465	10.722	√
78	FOXG_02330	alcohol dehydrogenase (NADP+)	42	DVDIKIECCGVCASD	8.173	2.412	√
			46	KIECCGVCASDRHTI	4.023	3.717	
79	FOXG_02339	tubulin beta chain	12	VHVQVGQCGNQVGSS	26.555	3.717	√
80	FOXG_02436	anthranilate synthase component I	2	******MCAAHLDIR	12.638	10.722	√
			294	PYVFFLSCADFHIVG	6.403	3.717	
81	FOXG_02845	high-affinity iron transporter	14	AVPIFFICFRECLET	27.194	3.717	√
			18	FFICFRECLETSIIV	12.716	10.722	
			268	KSVWHVNCCNAELNG	6.325	2.412	
82	FOXG_02932	probable xanthine phosphoribosyl transferase	16	YNDVHKMCQKSAEKL	30.303	3.717	√
83	FOXG_03138	ATP-dependent RNA helicase SUB2	2	******MCHTRELAY	40.75	3.717	√
84	FOXG_03244	catabolite repressor protein (CreC)	423	SYYGGLTCVCWSPDG	11.422	10.722	√
85	FOXG_03314	gluconate 5-dehydrogenase	10	FAPALRLCTRRVVAP	38.192	3.717	√

86	FOXG_03323	mitochondrial trans-2-enoyl-CoA reductase	6	**MSSSQCLRLRPLA	11.087	10.722	√
87	FOXG_03354	acyl-CoA synthetase-1	8	MYNIDWSCIQALVSD	12.786	10.722	√
			554	WLEVLLMCLLPGTPH	4.404	3.717	
88	FOXG_03391	Rab GDP-dissociation inhibitor	316	ADAADSSCIRTSRLT	11.081	10.722	√
89	FOXG_03411	NAD-dependent aldehyde dehydrogenase	20	LLTAWRRCNEFAAEH	12.06	3.717	√
90	FOXG_03423	casein kinase II subunit beta-1	138	YELGHFGCCPRTNCD	2.829	2.412	√
			139	ELGHFGCCPRTNCDQ	5.282	3.717	
91	FOXG_03447	mitochondrial 2-oxoglutarate/malate carrier protein	289	PYRSMMDCFSKVAKQ	5.532	3.717	√
92	FOXG_03509	hypothetical protein	180	WFFLVLMCATQDIAV	9.052	3.717	√
93	FOXG_03515	translation elongation factor	14	TGHLIYQCGGIDKRT	21.207	3.717	√
			392	MVPSKPMCVEAFTDY	4.202	3.717	
94	FOXG_03560	hypothetical protein	24	SVQYNSYCSARLSIR	4.257	3.717	√
95	FOXG_03575	Ras-like C3 botulinum toxin substrate 1	11	PGVQSLKCVVTGDGA	10.969	10.722	√
			196	SKQKKNKCSIL****	10.905	10.722	
96	FOXG_03707	ubiquitin carboxyl-terminal hydrolase 22/27/51	21	FLLPEINCWPGRKRK	11.664	3.717	
			105	QVPHCLSCAMDDMFQ	5.552	3.717	√
			259	CEYRCNNCNGTQQAK	4.259	3.717	
97	FOXG_03721	1,3-beta-glucan synthase component FKS1	362	RFMPECLCFIFKCAD	4.204	3.717	
			1603	AAMFGMACCMGPLLS	5.973	2.412	√
			1604	AMFGMACCMGPLLSM	9.019	3.717	
			1613	GPLLSMCCKKFGSVL	8.162	3.717	
98	FOXG_03729	putative coatomer subunit alpha, partial	146	GHNHYAMCAQFHPKE	4.096	3.717	√
99	FOXG_04556	related to alcohol/sorbitol dehydrogenase	116	CKRGLVNCCDKNGFI	7.686	2.412	√
100	FOXG_03731	SKT5 protein	914	QGKQDSDCILM****	13.605	10.722	√
101	FOXG_03746	trehalose 6-phosphate synthase	307	YSRHFLSCCTRILGF	11.357	2.412	√
102	FOXG_03787	succinate-CoA ligase alpha and beta chain	22	HLLRTAKCFSTSSAH	5.741	3.717	√

			162	TRLVGPNCPGIIAPN	5.058	2.412	
103	FOXG_03900	primary-amine oxidase	693	EEEAPSSCCATKTLP	10.91	2.412	√
			694	EEAPSSCCATKTLPR	6.277	3.717	
104	FOXG_04376	elongator complex protein 3	29	ESERFLRCCADVANA	13.076	2.412	
			30	SERFLRCCADVANAL	8.833	3.717	√
			111	IAVVAVMCKPHRCPH	5.37	3.717	
105	FOXG_04579	actin, gamma	17	LDNGSGMCKAGFAGD	33.556	3.717	√
106	FOXG_04841	phosphoribosyl-aminoimidazole carboxylase	20	GQLGRMLCEAASPLE	12.588	3.717	√
107	FOXG_05136	phospholipid methyltransferase	13	TLKELIECLTGPYLF	14.344	10.722	
			204	IVTILCLCSIPEPEK	4.221	3.717	√
			260	WSQVMGSCRICRSTG	5.081	3.717	
108	FOXG_05185	formate-tetrahydrofolate ligase	8	MYGAVLRCAGWLESS	37.35	3.717	√
			189	KSIREKLCAEVTEKQ	4.826	3.717	
109	FOXG_05187	adenosinetriphosphatase	458	IVMQLRKCCNHPYLF	11.112	2.412	√
			459	VMQLRKCCNHPYLFE	4.915	3.717	
110	FOXG_05207	aconitate hydratase, mitochondrial	127	AILQFMSCNLAKPAI	6.151	3.717	√
111	FOXG_05248	hypothetical protein	16	LRPLRRRCWQHPASG	28.833	3.717	√
			390	FFQALRLCETPCHRP	4.004	2.412	
112	FOXG_05335	imidazole glycerol phosphate synthase hisHF	465	AGEILLNCIDKDGTN	4.328	3.717	√
113	FOXG_05341	glutamate-cysteine ligase	274	MAFGMGSCCLQITFQ	7.711	2.412	√
			275	AFGMGSCCLQITFQA	5.346	3.717	
114	FOXG_05424	hypothetical protein	10	SRAAWRLCGRSQNTR	36.617	3.717	√
115	FOXG_05438	R3H domain protein, putative	309	LPPSNSFCRMLTHKL	3.963	3.717	√
116	FOXG_05445	delta-aminolevulinic acid dehydratase	175	RFPQLFICCDVCLCE	11.855	2.412	√
			176	FPQLFICCDVCLCEY	5.113	3.717	
117	FOXG_05773	hypothetical protein	145	RPQLVSMCKYMNLNT	4.898	3.717	√

118	FOXG_05833	3-hydroxy-3-methylglutaryl-coenzyme A reductase	795	WERVFGACCENVIGY	4.089	2.412	√
119	FOXG_05937	23S rRNA (-2'-O)-methyltransferase	49	VTRVVDLCAAPGSWS	4.602	3.717	√
			206	AALNLALCVLKPGGK	4.453	3.717	
120	FOXG_06093	hypothetical protein	8	MVTLLGFCFILINVA	40.213	3.717	
			17	ILINVAFCAIWMPDL	29.05	3.717	√
			329	WLVVTSICNYLGINA	4.35	3.717	
121	FOXG_06129	phosphoribosylformylglycinamidin	10	HVLIGESCYTASEVQ	38.167	3.717	√
122	FOXG_06160	elongation factor 1-alpha	308	HTKDPISCGDTFTMK	11.145	10.722	√
123	FOXG_06171	hypothetical protein	322	PTYYCEGCGCSHVAF	12.49	10.722	√
124	FOXG_06200	acetyl-CoA acyltransferase	303	KILGKYVCASIVGVK	4.881	3.717	√
125	FOXG_06447	L-ornithine N5-oxygenase	511	SGVWLQGCCEGTHGL	3.586	2.412	√
126	FOXG_07652	SGT1 and CS domain protein	98	QYANADACCVYAMRL	4.814	2.412	√
127	FOXG_07679	hypothetical protein	470	LDEFEAVCNEIHLEK	4.265	3.717	√
128	FOXG_07715	leucyl-tRNA synthetase, cytoplasmic	340	ETMYGQTCCFVGPKI	7.082	2.412	√
129	FOXG_07756	alpha	381	LYAVSDVCLVSSTRD	4.105	3.717	√
130	FOXG_06226	ATP-binding cassette, subfamily F, member 3	718	VCKSLWVCDGGTVEK	4.174	3.717	√
131	FOXG_06228	beta-tubulin, partial	12	VHLQTGQCGNQIGAA	27.58	3.717	√
132	FOXG_06240	mitochondrial metallochaperone Sco1	150	VYFGFTRCPDICPEE	4.306	2.412	√
133	FOXG_06313	dynein heavy chain, cytoplasmic	1969	GRFTLVFCCDDTFDF	8.466	2.412	√
			1970	RFTLVFCCDDTFDFQ	4.027	3.717	
134	FOXG_06351	protein pyrABCN	416	FINTMANCAEKPELL	5.752	3.717	√
			778	NSVTKSTCACFEPSL	3.883	3.717	
135	FOXG_06385	ferrochelatase	11	RRSGGQLCKSISQAS	12.552	10.722	√
			274	NRGDPLPC*******	12.771	10.722	
136	FOXG_07799	transformation/transcription domain-associated protein	11	IPLLFTRCSSRRFPH	35.7	3.717	
			47	EATSPSFCGMLLQFL	13.02	10.722	

			428	STFLLNVCKHFAIIE	3.728	3.717	√
			907	FKERLIDCFLGLEEK	12.389	10.722	
			2272	LHDASVICQSLANTN	4.195	3.717	
137	FOXG_07808	argininosuccinate synthase	7	*MSKGRVCLAYSGGL	15.426	10.722	√
138	FOXG_07867	diatom spindle kinesin 1	335	DPAAERSCKTVIVAC	12.648	10.722	√
139	FOXG_07877	phosphoglycolate phosphatase	11	INTLLFDCDNTLVLS	13.844	10.722	√
140	FOXG_07914	high-affinity iron transporter	191	VVGLFAGCCVGYLLY	10.635	2.412	
			192	VGLFAGCCVGYLLYK	4.626	3.717	√
			266	QSVWHVNCCASTEAI	5.157	2.412	
			267	SVWHVNCCASTEAIQ	3.729	3.717	
141	FOXG_07921	ribonucleotide reductase large subunit RNR1	352	NGDWTLMCPNECPGL	3.47	2.412	√
			876	SIEDPESCIMCSG**	12.843	10.722	
142	FOXG_07927	aminotransferase, classes I and II, putative	434	RDLFDSPCHHFVRLS	12.236	10.722	√
143	FOXG_07945	integral membrane protein	42	PRAFRPLCCIKTRLF	6.686	2.412	
			43	RAFRPLCCIKTRLFP	5.105	3.717	√
			56	FPNYKRTCCVPSSPY	4.978	2.412	
144	FOXG_08190	mitochondrial import inner membrane translocase	8	MDHGRDPCPWVILND	14.406	10.722	√
145	FOXG_08213	hypothetical protein	576	EGVLKVLCCTATLAW	7.053	2.412	√
		DEAD/DEAH box helicase, putative	577	GVLKVLCCTATLAWG	4.479	3.717	
146	FOXG_08214	synaptobrevin like ykt6	193	QAKKQNSCCILM***	14.039	10.722	√
			194	AKKQNSCCILM****	13.895	10.722	
147	FOXG_08237	hypothetical protein	4	****MSDCTTSTPNG	18.144	10.722	√
			132	VINMMVFCLQPWGEW	4.665	3.717	
148	FOXG_08241	serine/threonine-protein phosphatase	39	WIESLMSCKQLAEAD	6.852	3.717	√
149	FOXG_08257	DNA topoisomerase II	17	SFLLSRLCTRASPLA	32.267	3.717	√
150	FOXG_08280	hypothetical protein	509	ICERVCRCWRNMVIA	11.501	10.722	√

151	FOXG_08303	IST2 protein	405	VAWPLAACCFLVNNW	9.417	2.412	√
			406	AWPLAACCFLVNNWV	6.381	3.717	
152	FOXG_08306	tubulin-folding cofactor D (chaperone)	671	ERHGLLLCFASVLDK	3.745	3.717	√
			773	LVSALDSCDEDRSET	5.655	3.717	
153	FOXG_08320	phosphomevalonate kinase	284	KGVVLRMCDVDCGSK	3.902	3.717	√
154	FOXG_08357	dihydropteroate synthase	80	ACASGGGCGSSGPQK	13.264	10.722	√
155	FOXG_08363	N2, N2-dimethylguanosine tRNA methyltransferase	386	GPSADRHCEHCGMKM	14.756	10.722	√
156	FOXG_08381	ubiquitin-activating enzyme E1	9	NRQFLKPCVFARLKP	13.38	10.722	√
157	FOXG_08456	T-complex protein 1 subunit alpha	405	RSVHDSLCAVKRTLE	4.205	3.717	√
158	FOXG_08491	translation initiation factor 2 subunit 1	7	*MAQNQHCRFYEEKY	36.216	3.717	√
159	FOXG_08574	putative ATP-dependent RNA helicase	9	FRSSLRRCATQARSA	39.369	3.717	√
160	FOXG_08835	omega-6 fatty acid desaturase (delta-12 desaturase)	443	MYRSARMCQWVEPSA	3.905	3.717	√
161	FOXG_09037	formyltetrahydrofolate deformylase	11	DHILTLSCPDKSGIV	37.503	3.717	√
162	FOXG_09062	mitochondrial large ribosomal subunit YmL35	4	****MSRCQAVMRPI	13.243	10.722	√
163	FOXG_09219	hydrolase-HD superfamily protein	6	**MTISVCMRLPQTI	40.274	3.717	√
			164	LPKCMKMCLIHDMAE	7.698	3.717	
164	FOXG_09260	DNA-directed RNA polymerase I subunit RPA2	1148	SIVRCRACAVKLDDA	4.221	3.717	√
165	FOXG_09274	translation initiation factor 3 subunit H	19	TFRAPKPCNTTRPFR	21.18	3.717	√
166	FOXG_09771	ABC transporter CDR4	337	GFTTPWHCCVALTAS	7.485	2.412	√
			338	FTTPWHCCVALTASG	4.162	3.717	
167	FOXG_09794	mannitol 2-dehydrogenase	417	TGPFRRLCFVAGAWF	2.421	2.412	√
168	FOXG_09903	acyl-CoA thioester hydrolase	331	QTFELAFCCAASFAH	5.436	2.412	√
			332	TFELAFCCAASFAHA	7.196	3.717	
169	FOXG_10250	ribosome biogenesis protein ERB1	759	SAGADGTCRLWA***	12.378	10.722	√
170	FOXG_10255	tRNA ligase	512	HSSAKLVCMNFKHDE	3.799	3.717	√
171	FOXG_10485	mitochondrial processing peptidase	8	MLRPFTQCLPTRARA	35.189	3.717	

			17	PTRARAPCSRVLGQA	11.737	10.722	√
			436	ANMLDVMCQELRALT	4.015	3.717	
172	FOXG_10487	rRNA biogenesis protein RRP5, putative	541	PIGAVVTCNIEKVLI	4.312	3.717	√
			698	SKRLVVSCKDPSAFG	3.774	3.717	
173	FOXG_11049	ATP dependent RNA helicase (Dob1), putative	670	IKFAIVPCLLTCIKA	2.639	2.412	√
			853	MCAAVMSCFIFDEKI	4.761	3.717	
174	FOXG_11168	ankyrin repeat protein nuc-2	901	PNFPVFLCNDLGREE	5.044	3.717	√
175	FOXG_11275	armadillo repeat protein	691	NGVVTVLCEHAHSQN	4.259	3.717	√
176	FOXG_11294	long-chain acyl-CoA synthetase	475	GNIGGPMCCVELCLE	3.248	2.412	√
			476	NIGGPMCCVELCLES	4.112	3.717	
177	FOXG_11305	myosin-1	719	NEKAVFSCRAEILEA	5.675	3.717	√
178	FOXG_11318	methionyl aminopeptidase	16	KECMGADCQNEAGSL	34.142	3.717	√
			179	YNNFPKSCCTSVNEV	4.488	2.412	
179	FOXG_11385	acetyl-CoA acyltransferase	400	KMLLTSMCIGTGQGM	4.117	3.717	√
180	FOXG_11416	phosphatidate cytidylyltransferase	8	MQFFTLLCLATSALA	39.662	3.717	√
			7	*MALTGSCMCGAIAY	41.998	3.717	
181	FOXG_11429	seryl-tRNA synthetase	412	ATERTLCCILENYQT	4.619	3.717	√
182	FOXG_11436	succinate-semialdehyde dehydrogenase (NADP+)	345	RSPRQQICCRQGQGA	4.985	2.412	√
			401	GRDIRSPCPCL****	16.97	10.722	
183	FOXG_11474	hypothetical protein	16	LPALLTLCAIGVQAD	29.369	3.717	√
184	FOXG_11716	cyclopropane-fatty-acyl-phospholipid synthase	318	EEQSRLLCCDYRDIP	7.47	2.412	√
			319	EQSRLLCCDYRDIPE	5.117	3.717	
185	FOXG_11727	polyol transporter protein 1	199	GEIFITACCLTATPI	6.097	2.412	
			200	EIFITACCLTATPIA	5.579	3.717	√
			447	LITFPFMCVFLLAAG	4.01	3.717	
186	FOXG_12364	hypothetical protein	16	LFSLWLLCLGVLAHD	32.22	3.717	√

187	FOXG_12683	protein arginine N-methyltransferase 5	779	CSSRQVACLM*****	11.249	10.722	√	
188	FOXG_12719	hypothetical protein	535	TGLLGERCCGVGLLI	6.594	2.412	√	
			536	GLLGERCCGVGLLIE	6.902	3.717		
189	FOXG_12727	cleavage and polyadenylylation specificity factor	5	***MFTFCPLQGALS	41.982	3.717	√	
			73	HLAAYAHCCKNIPQF	4.375	2.412		
190	FOXG_12736	glutaryl-CoA dehydrogenase	9	FRPILRQCARRAAAP	38.635	3.717	√	
			20	AAAPLRPCATRTFAS	13.964	3.717		
191	FOXG_12747	hypothetical protein	430	KWAAAAMCLAKALVD	5.254	3.717	√	
192	FOXG_12773	ribose-phosphate pyrophosphokinase	21	PQLTENVCQILGVPA	11.127	3.717	√	
193	FOXG_13228	hypothetical protein	235	FSEAYRVCKPGAWIE	4.066	3.717	√	
195	FOXG_13297	hypothetical protein	20	GIGYGIACRIAANSP	9.954	3.717	√	
196	FOXG_13689	hypothetical protein	384	IPGSGSGCCIRLEAS	10.865	2.412	√	
			385	PGSGSGCCIRLEASL	4.131	3.717		
197	FOXG_15753	fumarylacetoacetase	212	LEMGMFICRENEMGS	4.277	3.717	√	
198	FOXG_13797	argininosuccinate synthase	7	*MAPERVCLAYSGGL	15.889	10.722	√	
199	FOXG_14464	hypothetical protein	21	TELIEAWCIDFGAIK	10.698	3.717	√	
200	FOXG_18811	hypothetical protein	374	PDSDLCDCMVKSRSY	4.152	3.717	√	
201	FOXG_17784	uroporphyrinogen decarboxylase	52	GNRDFFECCRDPEVA	6.274	2.412	√	
			53	NRDFFECCRDPEVAT	5.077	3.717		
202	FOXG_14637	L-lactate dehydrogenase	239	ASVAANLCCSVILDK	7.256	2.412	√	
			240	SVAANLCCSVILDKN	4.763	3.717		
203	FOXG_09473	eukaryotic translation initiation factor 5	121	DDRILLDCKACGQRT	5.228	3.717		√
204	FOXG_06194	hypothetical protein	15	PSSMASECKKCGKIL	27.821	3.717		√
			18	MASECKKCGKILTSF	11.599	10.722		
205	FOXG_18310	related to ferric reductase	364	AHKLEGACCCNSIQL	11.175	2.412		√
			365	HKLEGACCCNSIQLV	12.037	10.722		

			366	KLEGACCCNSIQLVV	5.563	3.717	
			519	AKGPTTGCCCSANGP	4.395	2.412	
			521	GPTTGCCCSANGPDE	4.166	3.717	
206	FOXG_08006	glyceraldehyde 3-phosphate dehydrogenase	23	DIISNASCTTNCLAP	9.884	3.717	√
207	FOXG_10630	cellular nucleic acid-binding protein	9	SSLSRRACYKCGNVG	34.802	3.717	√
			12	SRRACYKCGNVGHYA	13.906	10.722	
			22	VGHYAEVCSSAERLC	8.56	3.717	
208	FOXG_15088	hypothetical protein	199	KGVKWLSCMIQATGE	4.178	3.717	√
209	FOXG_15238	FMN hydroxy acid dehydrogenase	357	LINEFKLCMGLAGCS	4.256	3.717	√
210	FOXG_03392	exosome complex exonuclease RRP43	287	SVTVVVDCKDGDVKI	3.892	3.717	√
211	FOXG_00072	eukaryotic translation initiation factor 3 subunit D	11	SLDYVKICESCPAGD	32.92	3.717	
			14	YVKICESCPAGDGWG	29.253	3.717	√
			274	SILSMLMCSPRSVYP	4.98	3.717	

TABLE S2 Primers used in this study.

Purpose	gene ID	Primer names	Sequence (5’-3’)
**Prokaryotic expression**	FOXG_00780	FonAP-2α-GST-F	GATCTGGTTCCGCGTGGATCCATGGCGTGCATCAAGAGATGTCGAGCTCAGGC
		FonAP-2α-GST-R	GATGCGGCCGCTCGAGTCGACCATTCTGAAATTCTGCCTCGAGTCACCATAAC
	FOXG_08330	FonAP-2β-His-F	GCCATGGCTGATATCGGATCCATGGCGTGCATCAAGAGATGTTAAATAGGTGC
		FonAP-2β-His-R	GTGGTGGTGGTGGTGCTCGAGCATTCTGAAATTCTGCCTCGTAGCACTAACAA
	FOXG_04448	FonAP-2μ-GST-F	GATCTGGTTCCGCGTGGATCCATGGCGTGCATCAAGAG
		FonAP-2μ-GST-R	GATGCGGCCGCTCGAGTCGACCATTCTGAAATTCTGCCTCG
**Gene knockout and identification of knockout mutant**	FOXG_11304	FonPAT1-5'UTR-F	CATCAGGGTCTTCTGGTGGT
		FonPAT1-5'UTR-R	CAAAATAGGCATTGATGTGTTGACCTCCCTGGAGATACAGTGGTTCAAT
		FonPAT1-3'UTR-F	CGTCCGAGGGCAAAGGAATAGAGTAGTCCATCAGTCCATCCCAGAA
		FonPAT1-3'UTR-R	CGAGAAAACGAGCTCACCAT
		FonPAT1-ID1-F	GATGTGTGTTCTGCCCCTTT
		FonPAT1-ID1-R	ATCCAGGTTGGTGGTTAGCC
		FonPAT1-ID2-F	CAACTTCTTTACCACTGTCG
		FonPAT1-ID2-R	AATCCTTGGGTCCTTTATCC
	FOXG_06235	FonPAT2-5'UTR-F	CATAAACTTGCCTGCTTGTG
		FonPAT2-5'UTR-R	CAAAATAGGCATTGATGTGTTGACCTCCGAGATATAGCCCGTCATTGG
		FonPAT2-3'UTR-F	CGTCCGAGGGCAAAGGAATAGAGTAGGCTCGGTATCTCAACTCTCA
		FonPAT2-3'UTR-R	TTGTTTGGTTTCTTCGCC
		FonPAT2-ID1-F	GACTTACATCTACCCCAACA
		FonPAT2-ID1-R	GCTCACTCTTGATGAACTCG
		FonPAT2-ID2-F	TGTGGTGGTTACCGTGTTTC

	FonPAT2-ID2-R	CATTGGGTTCGTCATTTTTG
	FonPAT3-5'UTR-F	ACCCCAAGGTCAATGGAGGA
	FonPAT3-5'UTR-R	CAAAATAGGCATTGATGTGTTGACCTCCGAGGAGAAGATAGCAGCAGGC
	FonPAT3-3'UTR-F	CGTCCGAGGGCAAAGGAATAGAGTAGATTTGGAGGGTTATGTTTTC
	FonPAT3-3'UTR-R	TGCTGTCTGACTTTGGATCG
FOXG_07613	FonPAT3-ID1-F	TATCGTGAGCAAATACAGTC
	FonPAT3-ID1-R	AAGGGGTGTTGGTGTTGTAG
	FonPAT3-ID2-F	GTTTGGGTGGTGGTCTGTAT
	FonPAT3-ID2-R	GAGGTCAAAGACATTAGGGA
	FonPAT4-5'UTR-F	CTGGGGGCAACGGTGTAT
	FonPAT4-5'UTR-R	CAAAATAGGCATTGATGTGTTGACCTCCAGTGAAGGATGGAGGAAGGG
	FonPAT4-3'UTR-F	CGTCCGAGGGCAAAGGAATAGAGTAGATATGTGATATTCACGGGAA
	FonPAT4-3'UTR-R	TTAGACTGAGGTACTTTGCG
FOXG_02420	FonPAT4-ID1-F	AAGCAGAAAGGGGATAAGGG
	FonPAT4-ID1-R	GGCAAAAGGCTGAATGAGGA
	FonPAT4-ID2-F	TTGGGATGATGGAGGATTTC
	FonPAT4-ID2-R	TGAAGCCTTGCTCGTCTGTC
	FonPAT5-5'UTR-F	ACGATTTACAAGGAATGACG
	FonPAT5-5'UTR-R	CAAAATAGGCATTGATGTGTTGACCTCC TAAGCAAGGTAGGTAGGGAG
	FonPAT5-3'UTR-F	CGTCCGAGGGCAAAGGAATAGAGTAG TGGAAATAAACAAGGATAGGC
FOXG_03335	FonPAT5-3'UTR-R	TCGGTAAGAGCAGGAAGGAG
	FonPAT5-ID1-F	AGTTTGACGGATAGTGTTCG
	FonPAT5-ID1-R	GACTCGGCTTATGAGGGAC
	FonPAT5-ID2-F	ACAGAGCATTTTGATTACGG

	FonPAT5-ID2-R	CATCGTCTATCGTTTTCTTC
	FonPAT6-5'UTR-F	TAGCACATCACAAAGACGAC
	FonPAT6-5'UTR-R	CAAAATAGGCATTGATGTGTTGACCTCC TGTAACTACCCAATCAGGAAA
	FonPAT6-3'UTR-F	CGTCCGAGGGCAAAGGAATAGAGTAG CCACTGTGCCTTGCTTGAAT
	FonPAT6-3'UTR-R	TTGTGTTGTCTGGCGTTATC
FOXG_03646	FonPAT6-ID1-F	TGGAGGAGACAATGACAGCC
	FonPAT6-ID1-R	ACAACCACCTATCCCATCCC
	FonPAT6-ID2-F	CAGCACAAACAACAGAAGAT
	FonPAT6-ID2-R	TGAGAAGGAGGAGGATAGAA
	FonAP-2α-5'UTR-F	CCAGGTCAAAAGAAGCAGGC
	FonAP-2α-5'UTR-R	CAAAATAGGCATTGATGTGTTGACCTCCCAACGAACGACACAGAAGGC
	FonAP-2α-3'UTR-F	CGTCCGAGGGCAAAGGAATAGAGTAGTTCTCAGTCTCAGCGGTAGT
	FonAP-2α-3'UTR-R	TAGTTCCGTCAAAGAGCGTA
FOXG_00780	FonAP-2α-ID1-F	CTCACCTCCTCTTCCACC
	FonAP-2α-ID1-R	GCTCCTACTTCCCATACTCA
	FonAP-2α-ID2-F	GACGGCAGTCTTAGCGGTTAC
	FonAP-2α-ID2-R	ATTGCGGCGATACGATGT
	FonAP-2β-5'UTR-F	AGTCCTGGGTGGTATTCTGA
	FonAP-2β-5'UTR-R	CAAAATAGGCATTGATGTGTTGACCTCCCATCGCACGAGGTTATTAGC
	FonAP-2β-3'UTR-F	CGTCCGAGGGCAAAGGAATAGAGTAGACCAGTAAACCTAACCGAAC
FOXG_08330	FonAP-2β-3'UTR-R	GATAAAACGCTCAGACATAG
	FonAP-2β-ID1-F	GCTGTCCCATTGGTCCTT
	FonAP-2β-ID1-R	GTTTGCTTGGTCCCGTTT
	FonAP-2β-ID2-F	GCCTTCCCGTCCTTCTAT

		FonAP-2β-ID2-R	CGATGTTGTCCTCGTTGG
		FonAP-2μ-5'UTR-F	GACATTTGTGAGCGTTTG
		FonAP-2μ-5'UTR-R	CAAAATAGGCATTGATGTGTTGACCTCCCCATCTTTCTCCCTTCG
		FonAP-2μ-3'UTR-F	CGTCCGAGGGCAAAGGAATAGAGTAGGGGCGGTTTCTTTAGC
		FonAP-2μ-3'UTR-R	CGACCCAAGCGTGTAT
	FOXG_04448	FonAP-2μ-ID1-F	TCAGGCGTTCTGGTCT
		FonAP-2μ-ID1-R	GCTAGGCTCGTACTTGG
		FonAP-2μ-ID2-F	CAATCGCTTAGGGTGG
		FonAP-2μ-ID2-R	GGATGGGAGGAGGAAC
		FonAP-2σ-5'UTR-F	CATCATAGGGTAGTAGGTG
		FonAP-2σ-5'UTR-R	CAAAATAGGCATTGATGTGTTGACCTCCCTGAAAGTTGGGAGTTG
		FonAP-2σ-3'UTR-F	CGTCCGAGGGCAAAGGAATAGAGTAGCCGTGAGAATAGGTGG
		FonAP-2σ-3'UTR-R	GGTTTCGTTTGTGGACT
	FOXG_08592	FonAP-2σ-ID1-F	TAAAGGCGAAGTCCAT
		FonAP-2σ-ID1-R	CTCCAACCGTGTAAGC
		FonAP-2σ-ID2-F	TCGGCATACGACGCTAA
		FonAP-2σ-ID2-R	GCCCATACCACCCTCT
**HPH fragment**		HPH-F	GGAGGTCAACACATCAATGCCTATT
		HPH-R	GGAGGTCAACACATCAATGCCTATT
	FOXG_11304	FonPAT1-RT-F	GAAACCCCGCCATCGCTAA
		FonPAT1-RT-R	CGTGCAGCTCGACGAATCTTG
	FOXG_06235	FonPAT2-RT-F	TATGGAAACCCCGAGGTATGAC
		FonPAT2-RT-R	CCCCAAACCAATGGAGACG
	FOXG_07613	FonPAT3-RT-F	ACTACCGCTGTCTTTACACCCC

**qPCR**		FonPAT3-RT-R	ATCGAAATTCGCCGTTACTCTT
	FOXG_02420	FonPAT4-RT-F	ATGCGGTTTCAACGAGGACG
		FonPAT4-RT-R	GCACTCGAAACAGCCATAAGCA
	FOXG_03335	FonPAT5-RT-F	GTCCGCATCTTCACCACAGTTC
		FonPAT5-RT-R	CAGCGACCACCGAGACAGAA
	FOXG_03646	FonPAT6-RT-F	TAATCAACGAGCGAGCAACGG
		FonPAT6-RT-R	GGAGCACCACTTAGGGAAACCA
	FOXG_00780	FonAP2-α-RT-F	CCGACTGTACGCATCAGCTACC
		FonAP2-α-RT-R	GCACCACCAATCTGCTTCCAT
	FOXG_08330	FonAP2-β-RT-F	ATCCAAGAAGCCGAAACCCG
		FonAP2-β-RT-R	CCTCCTCCAAAGACATCATCCC
	FOXG_04448	FonAP2-μ-RT-F	CGAGCGGAAATAAGATGGGTAC
		FonAP2-μ-RT-R	CGGGAGGAACGAAGCTAATGAT
	FOXG_08592	FonAP2-σ-RT-F	GCGACGAGCAAAAGATCAAGC
		FonAP2-σ-RT-R	GCGTAGCGGCGGTAAACAAT
	FOXG_01569	FonActin-RT-F	GAGGGACCGCTCTCGTCGT
		FonActin-RT-R	GGAGATCCAGACTGCCGCTCAG
	EU603504	FonOpm12-RT-F	CGATTAGCGAAGACATTCACAAGACT
		FonOpm12-RT-R	ACGGTCAAGAAGATGCAGGGTAAAGGT
	HM036339	ClRps10-RT-F	AGGCTCACCCTAAAAGAAGG
		ClRps10-RT-R	GGTCAACACAAGGATCTTACT
	AB266616	FonFow2-RT-F	AAGAAGTCTGGCTCTAGTGGAA
		FonFow2-RT-R	GCAGAAGGCAAGGAATAAGGAA
	FOXG_06120	FonSho1-RT-F	CCTCGAAGTGTCGGATGTTAGC

		FonSho1-RT-R	GCATGGATCGTTGCTCATAACA
		FonFvs1-RT-F	CCACGTCATCGGAAACAGAAA
		FonFvs1-RT-R	CGCGAAGTGAATGACCAGAAC
		FonMsb2-RT-F	TCGTCCGTAATCTGACCTCCG
		FonMsb2-RT-R	GTTGCCATCATCCGAGTTACCAT
**Construction of mCherry or GFP-fusion cassettes**	FOXG_11304	FonPAT1-mcherry-F	GCATGGACGAGCTGTACAAGATGGCCGGCCTCAACGACGT
		FonPAT1-mcherry-R	AGCATTGATGTGTTGACCTCCATCGTCTGAAATATCTCGCT
		FonPAT1-promoter-F	ATCTCATGAAGGCGTGTAGG
		FonPAT1-promoter-R	ATCCTCCTCGCCCTTGCTCACCATGATGAAGCATTAACTTGAGC
	FOXG_06235	FonPAT2-mcherry-F	GCATGGACGAGCTGTACAAGATGGTTGTGGGCGAGTATAT
		FonPAT2-mcherry-R	AGCATTGATGTGTTGACCTCCCCGCCTGCGCCGTCTCTCGG
		FonPAT2-promoter-F	TTGTCTTGGGGCTTGTACGG
		FonPAT2-promoter-R	ATCCTCCTCGCCCTTGCTCACCATAATGAGCATGAAGAAAATCT
	FOXG_02420	FonPAT4-mcherry-F	GCATGGACGAGCTGTACAAGATGGGGAGCATCAGTACCAG
		FonPAT4-mcherry-R	AGCATTGATGTGTTGACCTCCAACCTCTTCCGTTCCAACGG
		FonPAT4-promoter-F	GTCTGCACCAGGACCATCGC
		FonPAT4-promoter-R	ATCCTCCTCGCCCTTGCTCACCATGGCAGGCTGGCTTGTAGCGC
	FOXG_00780	FonAP-2α-mcherry-F	CTCGGCATGGACGAGCTGTACAAGATGTCGAGCTCAGGCACAGG
		FonAP-2α-mcherry-R	AGCATTGATGTGTTGACCTCCAGTCACCATAACATTACGGA
		FonAP-2α-promoter-F	ACTCACTATAGGGCGAATTGGGTACTCAAATTGGTTCCAGGTCAAAAGAAGC
		FonAP-2α-promoter-R	TTTGGGCGGTGGTATGCAAA
	FOXG_08330	FonAP-2β-mcherry-F	GCATGGACGAGCTGTACAAGATGTCGAGCTCCGCCGGTGG
		FonAP-2β-mcherry-R	AGCATTGATGTGTTGACCTCCTAGCACTAACAAATCACC
		FonAP-2β-promoter-F	ACTCACTATAGGGCGAATTGGGTACTCAAATTGGTTGCTGTCCCATTGGTCCTT

		FonAP-2β-promoter-R	GGTGTTGTACTTGTATCC
	FOXG_04448	FonAP-2μ-mcherry-F	GCATGGACGAGCTGTACAAGATGTTGTCAGGCGTTCTGGT
		FonAP-2μ-mcherry-R	AGCATTGATGTGTTGACCTCCAAATCTTATTTCATATGATC
		FonAP-2μ-promoter-F	ACTCACTATAGGGCGAATTGGGTACTCAAATTGGTTCAATCGCTTAGGGTGGT
		FonAP-2μ-promoter-R	GGTCGCGATTTGAGGGTGTT
	FOXG_08592	FonAP-2σ-mcherry-F	ACTCACTATAGGGCGAATTGGGTACTCAAATTGGTTTGATGGCGAGTGTATT
		FonAP-2σ-mcherry-R	CTTGAATTTGGCAAGGCTGA
		FonAP-2σ-promoter-F	GCATGGACGAGCTGTACAAGATGCTTTCCTTTATCCTCAT
		FonAP-2σ-promoter-R	AGCATTGATGTGTTGACCTCCTTCTAACTTGTCGAGATGCT
**Construction of pYES2 for yeast complementation**	FOXG_11304	FonPAT1-PYES2-F	CAGCTTATCATCGATAAGATGGCCGGCCTCAACGACGT
		FonPAT1-PYES2-R	AACCGCTAACAATACCTGCTAATCGTCTGAAATATCTC
	FOXG_06235	FonPAT2-PYES2-F	CAGCTTATCATCGATAAGATGGTTGTGGGCGAGTATAT
		FonPAT2-PYES2-R	AACCGCTAACAATACCTGCCGCCTGCGCCGTCTCTCGG
	FOXG_02420	FonPAT4-PYES2-F	CAGCTTATCATCGATAAGATGGGGAGCATCAGTACCAG
		FonPAT4-PYES2-R	AACCGCTAACAATACCTGCTAAACCTCTTCCGTTCCAA
**Amplification of fragments**	FOXG_11304	FonPAT1-C117S-F	CCAAGATGGATCATCATaGTCCTTGGACTAGGAAC
		FonPAT1-C117S-R	GTTCCTAGTCCAAGGACtATGATGATCCATCTTGG
	FOXG_06235	FonPAT2-C99S-F	CCAAGGCTGATCATCATaGCGTCTTTATAAATTCA
		FonPAT2-C99S-R	TGAATTTATAAAGACGCtATGATGATCAGCCTTGG
	FOXG_02420	FonPAT4-C475S-F	CCAAGCATGATCACCATaGTCCTTGGGTTTACAAC
		FonPAT4-C475S-R	GTTGTAAACCCAAGGACtATGGTGATCATGCTTGG
	FOXG_00780	FonAP-2α-C400S-F	TACAGCATGaGTGACGCCACAAATGCCCAAGT
		FonAP-2α-C400S-R	GCGTCACtCATGCTGTAGAGTAAGTCCAGACCC
	FOXG_08330	FonAP-2β-C5S-F	GGaGCTTCTTGTTTCTGGTCAACTATGCCAGG

**carrying point mutation**		FonAP-2β-C5S-R	CAGAAACAAGAAGCtCCTATTTAACATAAGCCTCTGAGCG
		FonAP-2β-C260S-F	GCAAGTACAACGATCCTATTTATGTCAAGGTCACAAAGCTGGAACTTATCTTC
		FonAP-2β-C260S-R	GATCGTTGTACTTGCtGAAAAAGACACGTATATCGTTTCGG
	FOXG_04448	FonAP-2μ-C24S-F	GGAACGACaGTCGTCCTCGCCTCGCAGACGTC
		FonAP-2μ-C24S-R	AGGACGACtGTCGTTCCGGAAGGCGCGGAAGA
**CoIP constructs**	FOXG_00780	FonAP-2α-GFP-F	ATACCTTTGCATACCACCGCCCAAAATGTCGAGCTCAGGCACAGG
		FonAP-2α-GFP-R	CACCACCCCGGTGAACAGCTCCTCGCCCTTGCTCACAGTCACCATAACATTAC
		FonAP-2α-GFP-ID-F	ACGACAGTCTGCACTTCTCT
		FonAP-2α-GFP-ID-R	TGGTAGTGGTCGGCGAGCTG
	FOXG_08330	FonAP-2β-FLAG-F	CTATAGGGCGAATTGGGTACTCAAATTGGTTGCTGTCCCATTGGTCCTTGT
		FonAP-2β-FLAG-R	CTTTATAATCACCGTCATGGTCTTTGTAGTCTAGCACTAACAAATCACCAT
		FonAP-2β-FLAG-ID-F	TTGATTCTCCAGCGACGACC
		FonAP-2β-FLAG-ID-R	TGGTAGTGGTCGGCGAGCTG
	FOXG_04448	FonAP-2μ-GFP-F	ACTCACTATAGGGCGAATTGGGTACTCAAATTGGTTCAATCGCTTAGGGTGGT
		FonAP-2μ-GFP-R	CACCACCCCGGTGAACAGCTCCTCGCCCTTGCTCACAAATCTTATTTCATATGA
		FonAP-2μ-GFP-ID-F	GAGGCCCGGTCCGAAAACAC
		FonAP-2μ-GFP-ID-R	TGGTAGTGGTCGGCGAGCTG
